# Can serum autoantibodies be a potential early detection biomarker for breast cancer in women? A diagnostic test accuracy review and meta-analysis

**DOI:** 10.1186/s13643-022-02088-y

**Published:** 2022-10-09

**Authors:** Thejas Kathrikolly, Sreekumaran N. Nair, Aju Mathew, Prakash P. U. Saxena, Suma Nair

**Affiliations:** 1 Present Address: Department of Community Oncology, Sri Shankara Cancer Hospital and Research Centre, Bengaluru, India; 2 Department of Community Medicine, Kasturba Medical College, Manipal Academy of Higher Education (MAHE), Manipal, India; 3 Department of Biostatistics, Jawaharlal Institute of Postgraduate Medical Education & Research (JIPMER), Puducherry, India; 4 Department of Oncology, MOSC Medical College Kolenchery, Kerala, India; 5 Department of Internal Medicine, University of Kentucky Markey Cancer Center, Lexington, USA; 6 Department of Radiation Oncology, Kasturba Medical College, Mangalore, India; 7 School of Public Health, DY Patil Deemed to be University, Navi Mumbai, India

**Keywords:** Breast cancer, Biomarkers, Autoantibodies, Early detection

## Abstract

**Background:**

The increasing incidence of breast cancer necessitates the need to explore alternate screening strategies that circumvent the setbacks of conventional techniques especially among population that report earlier age at diagnosis. Serum autoantibodies is one such potential area of interest. However, their ubiquitous presence across cancer types limits its applicability to any one specific type of cancer. This review was therefore carried out to explore and consolidate available evidence on autoantibodies for early detection of breast cancer and to identify those that demonstrated a higher sensitivity.

**Methods:**

A diagnostic test accuracy (DTA) review was carried out to ascertain serum autoantibodies that could be used for early detection of breast cancer among women. All relevant articles that investigated the role of autoantibodies in early detection of breast cancer were included for the review. MEDLINE, Scopus, ProQuest, Ovid SP, and Cochrane Library were searched extensively for eligible studies. Quality of the included studies was assessed using Quality Assessment of Diagnostic Accuracy Studies (QUADAS)-2 tool. RevMan 5.3 was used for exploratory and MetaDTA 2019 for hierarchical analyses. The review helped identify the most frequently investigated autoantibodies and a meta-analysis further consolidated the findings.

**Results:**

A total of 53 articles were included for the final analysis that reported over a 100 autoantibodies that were studied for early detection of breast cancer in women. P53, MUC1, HER2, HSP60, P16, Cyclin B1, and c-Myc were the most frequently investigated autoantibodies. Of these P53, MUC1, HER2, and HSP60 exhibited higher summary sensitivity measures. While the individual pooled sensitivity estimates ranged between 10 and 56%, the panel sensitivity values reported across studies were higher with an estimated range of 60–87%.

**Conclusion:**

Findings from the review indicate a higher sensitivity for an autoantibody panel in comparison to individual assays. A panel comprising of *P53*,* MUC1*,* HER2*, and *HSP60 *autoantibodies has the potential to be investigated as an early detection biomarker for breast cancer.

**Supplementary Information:**

The online version contains supplementary material available at 10.1186/s13643-022-02088-y.

## Background

According to GLOBOCAN 2020 estimates, breast cancer was the most commonly diagnosed cancer and accounted for around 2.3 million new cases worldwide. It has surpassed lung cancer as the most commonly diagnosed type of cancer [1]. Over the past decade, the scenario of breast cancer burden in India has become a cause for concern with available data suggesting it to be the foremost reason for female cancer deaths in the country [2]. For every two women newly diagnosed with breast cancer in India, one woman dies of it signifying a high mortality to incidence ratio [2]. Findings underscore that certain factors, particularly lack of awareness and poor access to effective screening methods, along with delayed diagnosis are likely significant contributors to the high mortality.

Most breast cancers are amenable to treatment if detected early enough [3]. Screening strategies that aid early detection is the key to reduce mortality rates. However, in the Indian setting where the median age of diagnosis of breast cancer is nearly a decade younger when compared to the western population, existing strategies such as mammography is seldom effective [2]. Clearly, there is a need to devise and implement alternate screening strategies that could circumvent the setbacks of conventional screening techniques. One such promising approach is the field of cancer immunology that has brought forth the potential of serum autoantibodies that could contribute as early detection markers for breast cancer.

Serum antibody against tumor-associated antigens may have a potential as an early detection strategy in breast cancer as it is detected in circulation even before the clinical manifestations of the disease. In addition, it is produced in considerable amount and remain in circulation for a longer period due to limited clearance [4]. Nonetheless, universal presence of certain autoantibodies across different types of cancers has limited its applicability in early detection of any one specific cancer.

This systematic review was carried out with an aim to ascertain serum autoantibodies that could be used for early detection of breast cancer among women. Since research on autoantibodies for early detection has consistently advocated a panel or a multiplex of autoantibodies rather than one autoantibody for enhanced sensitivity [3, 5], this systematic review also aimed at identifying an autoantibody multiplex comprising of the most sensitive autoantibodies.

Considering the setbacks of conventional screening modalities, findings of this diagnostic test accuracy review bears significance in the context of developing alternate early detection strategies, especially among young women in low-resource settings. The findings provide a basis for large-scale analytical studies to validate the application of serum autoantibodies in early detection of breast cancer.

## Materials and methods

We conducted a diagnostic test accuracy (DTA) review to identify specific autoantibodies and their role in early detection of breast cancer. The formulated research question for this review, adhering to the PICO guidelines was ‘When compared with healthy individuals, which serum antibody or antibodies against tumor antigens found in breast cancer patients can serve as biomarker for detecting breast cancer?’ As this is a diagnostic test accuracy review, certain components of PICO are different in comparison to interventional studies. Here, the population (P) comprised of breast cancer patients. The intervention (I) and comparison (C) referred to the index test (autoantibodies) and purpose of the index test (early detection), respectively. Lastly, the outcome (O) was the target disorder or breast cancer. We had two primary objectives for this review—(a) to compile available evidence on serum autoantibodies used for early detection of breast cancer and (b) to identify autoantibodies that have demonstrated a higher sensitivity in detecting early breast cancer.

The protocol was developed using the PRISMA-P (Preferred Reporting Items for Systematic review and Meta-Analysis Protocols) checklist [6]. We complied with all the 17 recommended items in the checklist. A framework of all the steps was put in place for easy reference and the review was carried out over a period of 1 year.

### Criteria for considering studies for this review

#### Inclusion criteria

Diagnostic test studies using serum autoantibodies for breast cancer diagnosis in women of any age group. ELISA (enzyme-linked immunosorbent assay) as the preferred method of analysis either standalone or in combination with other techniques such as phage display, bio-panning, 2D electrophoresis (2DE), western blot (WB), proteomic analysis, and mass spectrophotometry (MS).

#### Exclusion criteria

Studies were excluded if (a) they were animal studies (b) the sample used for analysis was not blood (c) samples were analyzed post-surgery or post-therapy, and (d) autoantibody detection was not applied for early detection/ diagnosis.

#### Search criteria

A search strategy was conceived using a combination of Medical Subject Headings (MeSH) and controlled vocabulary to identify peer-reviewed articles on our topic of interest. PubMed/MEDLINE, Scopus, Proquest, Ovid SP, and Cochrane Library were searched for relevant articles. Key words used were ‘breast’, ‘breast cancer’, ‘breast carcinoma’, ‘tumour associated antigens’, ‘antibodies’, ‘serum’, ‘biomarker’, ‘blood’, ‘screening’, ‘detection’, ‘early detection’, and ‘diagnosis’. MeSH terms for the keywords such as ‘breast neoplasm’, ‘neoplasm antigens’, ‘autoantibodies’, ‘carcinogen markers’, and ‘cancer screening’ were also utilized for expanding the search. Details of search strategy used in each database is described in Additional file 1: Table S1. These were combined with appropriate Boolean operators so as to generate relevant results. As the use of special filters like ‘diagnostic study’, ‘sensitivity’, and specificity’ for retrieval of diagnostic test studies may cause to overlook relevant studies, these were avoided [7, 8]. The search was updated till December 2020.

### Study selection and data extraction

Studies were initially screened for eligibility by their titles and abstracts. Full text of the eligible articles was retrieved and reviewed prior to including them in the final analysis. The process of screening each potentially relevant study for inclusion in the review was carried out independently by two authors Suma Nair (SN) and Thejas Kathrikolly (TK) using the eligibility form based on the inclusion criteria. We excluded studies that did not meet the eligibility criteria and listed the reasons for exclusion in the’Characteristics of excluded studies’ table (Additional file 2). Data from the selected studies were extracted using a data extraction form (Additional file 3). Disagreements were resolved through discussion with other co reviewers Sreekumaran Nair (SNN), Prakash Saxena (PU), and Aju Mathew (AM).

### Quality assessment of included studies

Full text articles of the selected studies were assessed for quality with the help of a tailored Quality Assessment of Diagnostic Accuracy Studies (QUADAS)-2 tool [7, 9] (Additional file 4).

### Data analysis

Primary exploratory analyses were carried out with RevMan 5.3 [10]. This included quality assessment summary of eligible studies, producing paired forest plots of sensitivity and specificity for autoantibodies and summary receiver operating characteristic (SROC) curves. We carried out meta-analysis for the common autoantibodies—P53, MUC1, HER2, HSP60, Cyclin B1, c-Myc, and P16. Since heterogeneity is inherent in DTA studies, hierarchical models were used for analysis viz., HSROC (Hierarchical Summary Operating Characteristic) curve to estimate pooled sensitivity and specificity. MetaDiSC version 1.4. and MetaDTA 2019, were used for the analysis [11].

## Results

**Fig. 1:**
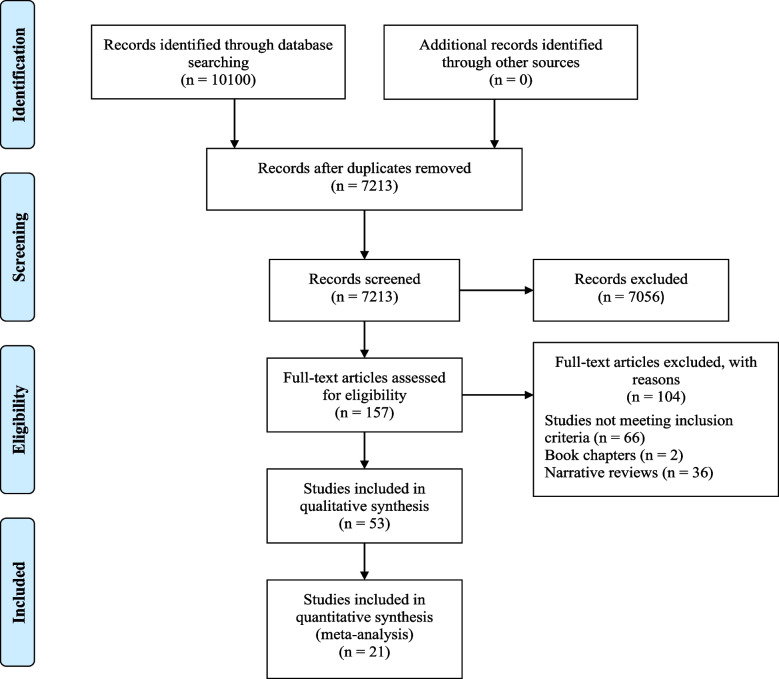
PRISMA chart with the search results

A total of 10,100 published articles were retrieved in all. After excluding the duplicates, 7213 articles were screened by their titles and abstracts to look for their relevance for inclusion. Of these, 7056 articles were rejected as they did not concur with our inclusion criteria. The remaining 157 articles were selected for full-text retrieval to check their eligibility to be included for the final analysis. Of these, articles that did not focus on early- stage patients or autoantibodies for early detection were excluded and, in the end, we had 53 articles that met the inclusion criteria of the review and were included for the final analysis. The PRISMA chart illustrating the search results is shown in Fig. 1.

### Characteristics of included studies

Details of the included studies are presented in Tables S2 and S3 (Additional file 5). Majority of the studies were diagnostic studies with a reversed flow design. Details about study design and population characteristics, type of sample used for analysis, detection techniques and autoantibodies investigated are tabulated in Table S1. Description of index tests, reference tests, definition of threshold, and diagnostic measures reported in each study are depicted in Table S2.

**Fig. 2:**
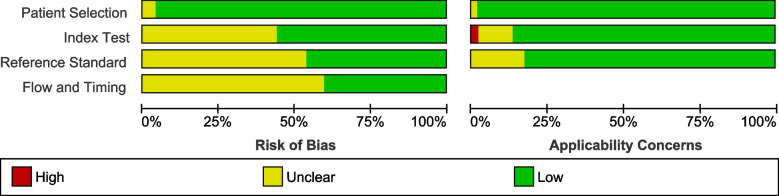
Risk of bias summary table (Methodological quality summary): review authors’ judgements about each methodological quality item for each included study

Study characteristics were further entered in RevMan 5.3 to generate a risk of bias summary table representing the quality of studies as illustrated in Fig. 2*.*

Studies were assessed based on four domains of the QUADAS-2 tool and showed low risk of bias and applicability concerns.

### Population included in the studies

Majority of the studies had recruited women barring three that reported male participants [12–14]. Five studies reported the role of autoantibodies in other types of cancers in addition to breast cancer [12, 15–18]. Almost all the diagnostic test studies used the case control study design except the one by Regele et al. that was cross sectional in nature [19].

### Tests, sample, and methods of analysis used in the studies

There was considerable variation among the studies with respect to index tests, reference tests, samples, and methods used for investigating autoantibodies. Biopsy proven diagnosis and sometimes radiological and clinical examination were used to confirm the breast cancer status of participants, while routine health check and follow-up, detailed medical history, routine mammograms, and radiological examination were the procedures used to group the participants as healthy as shown in (Table S2, Additional file 5).

Although our pre-defined inclusion criteria specified serum as the preferred sample for detecting autoantibodies, we also included studies that used plasma for analysis, as they contained significant information about autoantibodies for early detection [20–26]. One study by Yi et al. had used urine as a sample source of breast cancer tumor proteins in addition to serum [27].

Majority of the studies used ELISA as the method of analysis. However, many studies had combined ELISA with western blotting, immunohistochemistry, proteomics, and microarrays to gain better sensitivity at autoantibody detection as presented in (Table S2 Additional file 5).

### Autoantibodies

A total of 100 autoantibodies (Additional file 6**)** were investigated for their role in early detection and diagnosis of breast cancer. Most frequently studied autoantibodies were P53 [3, 14, 15, 19, 25, 28–38], MUC1 [3, 5, 39–42], HER2 [3, 32, 35], HSP60 [5, 43, 44], Cyclin B1 [35, 37, 38, 45], c-Myc [3, 34, 36, 38, 46], and P16 [21, 34, 37, 38, 47]. The diagnostic measures of individual autoantibodies were investigated by all studies. Ten studies also reported the measures for autoantibody panels [3, 5, 25, 32, 34, 36, 38, 42, 46, 48].

### Threshold

Studies adopted three common measures of threshold to define a positive value for autoantibodies, an absorbance value greater than the mean + 2, + 3, or + 4 standard deviations (SDs) of the normal cohort (Additional file 7)*.* Some studies reported a pre- defined threshold specific to the method of analysis [14, 15, 17, 20, 26, 28, 30, 31, 40, 49] while some others did not report any threshold for a positive cut-off [5, 12, 19, 21, 23, 24, 27, 29, 34, 42, 44–46, 48, 50–54].

### Reporting of diagnostic measures

Studies have reported the applicability of autoantibodies for early detection and diagnosis in the form of sensitivity, specificity, and other diagnostic measures like area under the curve (AUC) values. Efforts were made to extrapolate the percentage of autoantibody positivity in patient and healthy groups to derive sensitivity and specificity values with the help of 2 × 2 tables (Additional file 8). Three studies reported measures in odds ratio [22, 33, 51]. Results from studies that investigated individual autoantibodies in addition to autoantibody panels suggested that although the sensitivity values were less for individual autoantibodies, such autoantibodies when combined in a panel demonstrated higher sensitivity values.

Most frequently investigated autoantibodies were identified and their sensitivity measures when used as a stand- alone or when included in a multiplex were compared*.* These autoantibodies were P53, MUC1, HER2, HSP60, Cyclin B1, c-Myc, and P16. We found 16 studies that investigated the P53 autoantibody and another 8 that studied MUC1 autoantibodies in early detection of breast cancer. C-Myc and P16 were investigated by five studies each, while four studies investigated HER2, HSP60, and Cyclin B1 autoantibodies. Individual and panel values of these autoantibodies are illustrated in Table 1 that clearly illustrate a higher sensitivity for the panel.

**Table 1:** Comparison of individual and panel sensitivity values

*Autoantibody*	*Autoantibody panel (Sensitivity %)*	*Stand-alone Sensitivity %*
P53	*P53,* Cyclin B1, p16, p62, 14–3-3ξ (78)	3.4–47
	*P53,* HSP60, FKBP52, PRDX2, PP1A, MUC1, GAL3, PAK2, CCNB1, PHB2, RACK1, RUVBL1, HER2 (70–77)	
	*P53*, c-Myc, NY-ESO-1, BRCA-1, BRCA-2, HER-2, MUC1 (64)	
	*P53*, c‑Myc, survivin, cyclin, B1, cyclin D1, p62, p12, CDK2 (61)	
MUC1	*MUC1*, LGALS3, Phb2, GK2, CA 15–3 (87)	20–50
	*MUC1,* ANGPTL4, DKK1, EPHA2, GAL1, HER-2, IGFBP2, LAMC2, SPON2, CST2, SPINT2, SSR2 (73)	
	*MUC1,* HSP60, FKBP52, PRDX2, PP1A, P53, GAL3, PAK2, CCNB1, PHB2, RACK1, RUVBL1, HER-2 (70–77)	
	*MUC1,* P53, c-myc, NY-ESO-1, BRCA-1, BRCA-2, HER-2 (64)	
	*MUC1,* PPIA, PRDX2, FKBP52, HSP60 (61)	
HER2	*HER-2,* MUC1, ANGPTL4, DKK1, EPHA2, GAL1, IGFBP2, LAMC2, SPON2, CST2, SPINT2, SSR2 (73)	13–17
	*HER2,* HSP60, FKBP52, PRDX2, PP1A, MUC1, GAL3, P53, PAK2, CCNB1, PHB2, RACK1, RUVBL1 (70–77)	
	*HER-2,* c-Myc, NY-ESO-1, BRCA-1, BRCA-2, P53, MUC1 (64)	
HSP60	*HSP60*, FKBP52, PRDX2, PP1A, MUC1, GAL3, PAK2, CCNB1, PHB2, RACK1, RUVBL1, HER2, P53 (70–77)	33–48
	*HSP60,* MUC1, PPIA, PRDX2, FKBP52 (61)	
c-Myc	*c-Myc,* cyclin B1, Imp1, p16, Koc, survivin (67)	3.8–22
	*c-Myc,* NY-ESO-1, P53, BRCA-1, BRCA-2, HER-2, MUC1 (64)	
	*c‑Myc,* P53, survivin, cyclin, B1, cyclin D1, p62, p12, CDK2 (61)	
Cyclin B1	C*yclin B1*, P53, p16, p62, 14–3-3ξ (78)	12–18.4
	*Cyclin B1,* Imp1, P16, Koc, survivin, c-Myc (67)	
	*Cyclin B1,* c‑Myc, survivin, P53, cyclin D1, p62, p12, CDK2 (61)	
P16	*P16*, CyclinB1, P53, p62, 14–3-3ξ (78)	12.2–30.3
	*P16*, Cyclin B1, Imp1, Koc, survivin, c-Myc (67)	

### Meta- analysis

**Fig. 3:**
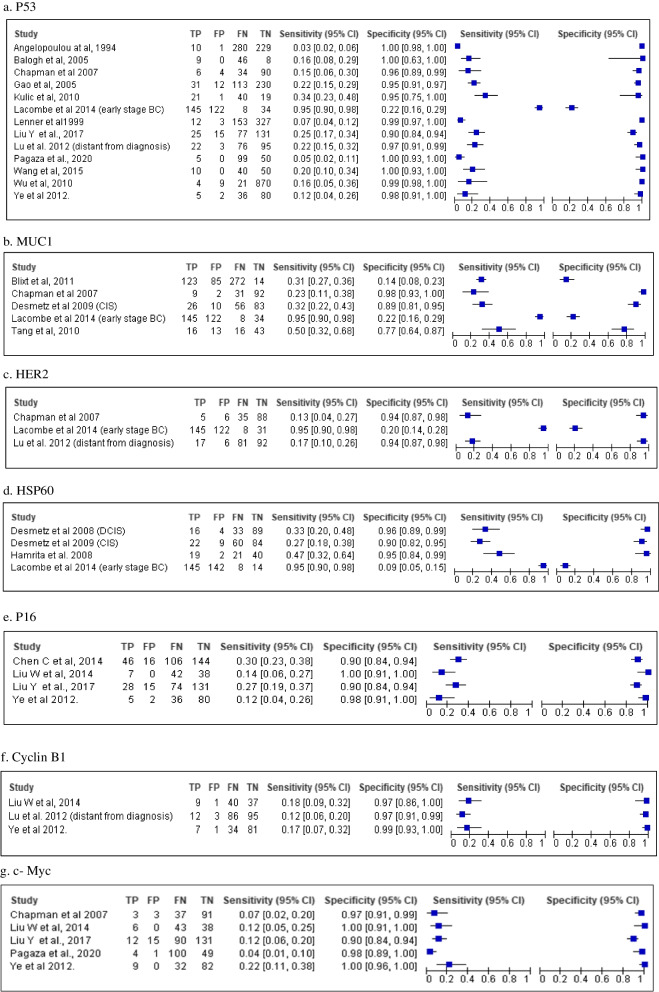
Coupled forest plots of autoantibodies

Coupled forest plots of sensitivity and specificity for the most frequently investigated autoantibodies, P53, MUC1, HER2, HSP60, Cyclin B, c-Myc, and P16 were developed using RevMan 5.3 and are depicted in Fig. 3. Their corresponding summary ROC (SROC) curves are shown in Additional file 9. This was the preliminary step in assessing heterogeneity of the studies to guide further hierarchical analysis.

**Fig. 4:**
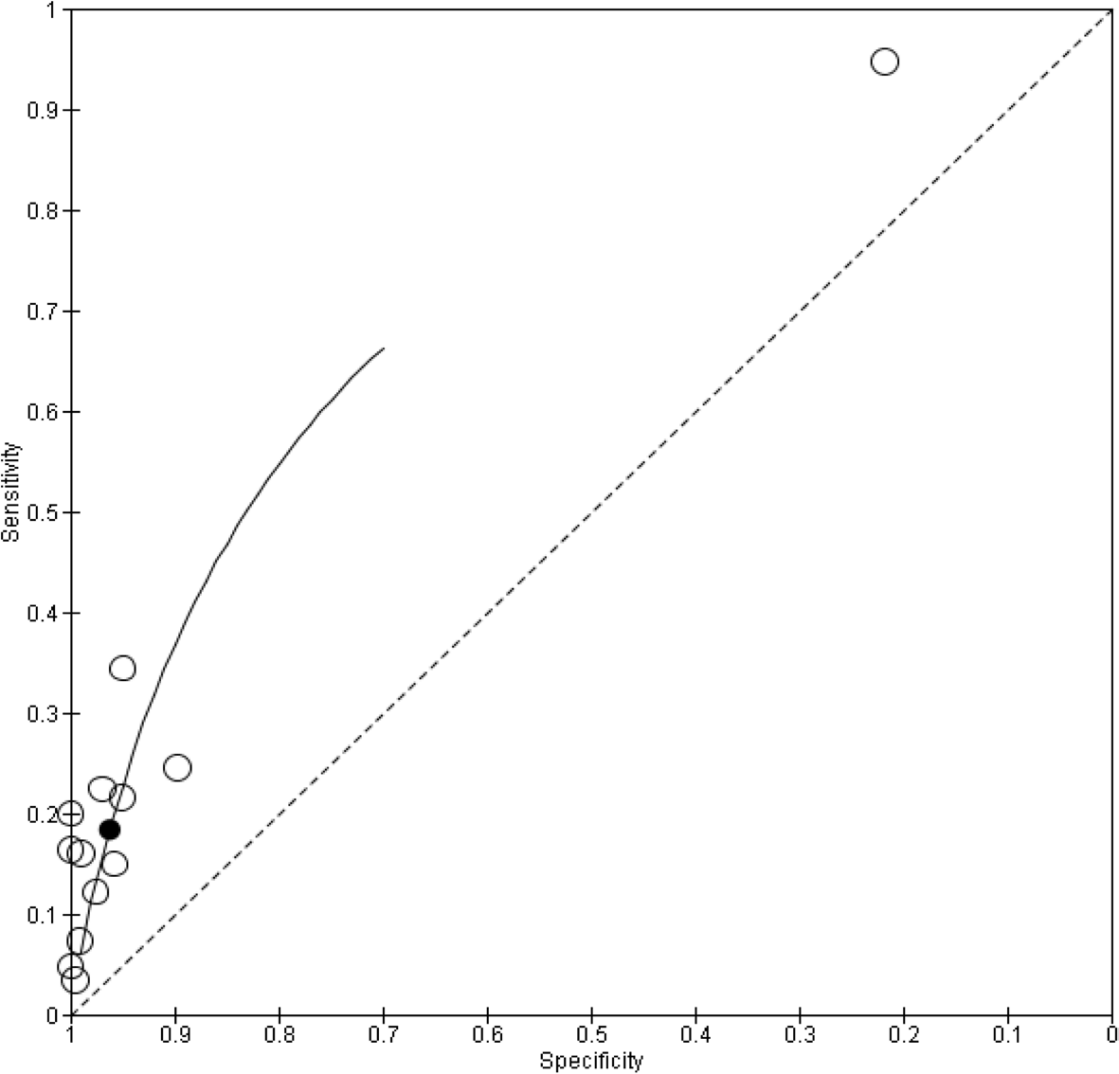
HSROC curve for P53

**Fig. 5:**
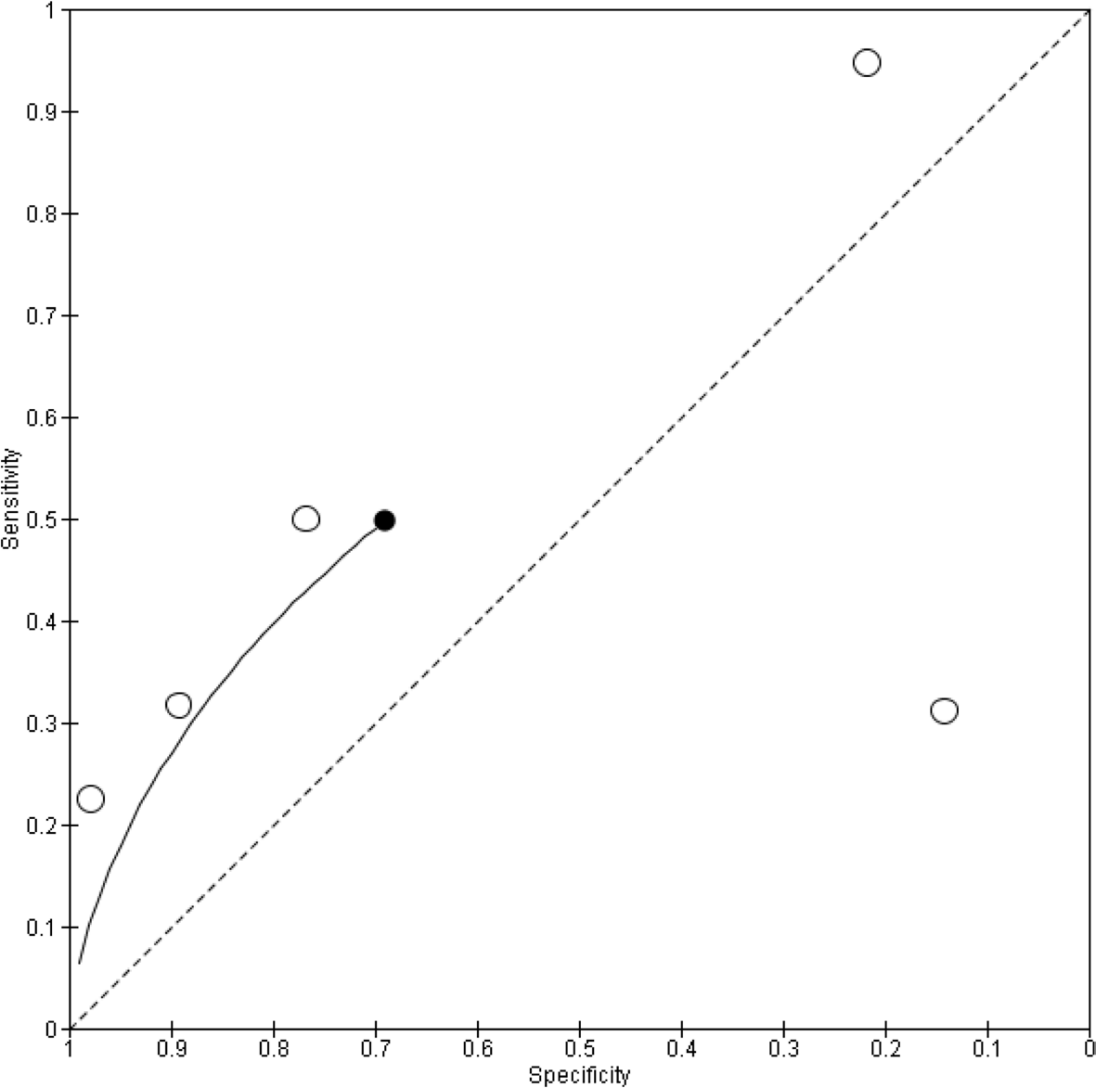
HSROC curve for MUC1

**Fig. 6:**
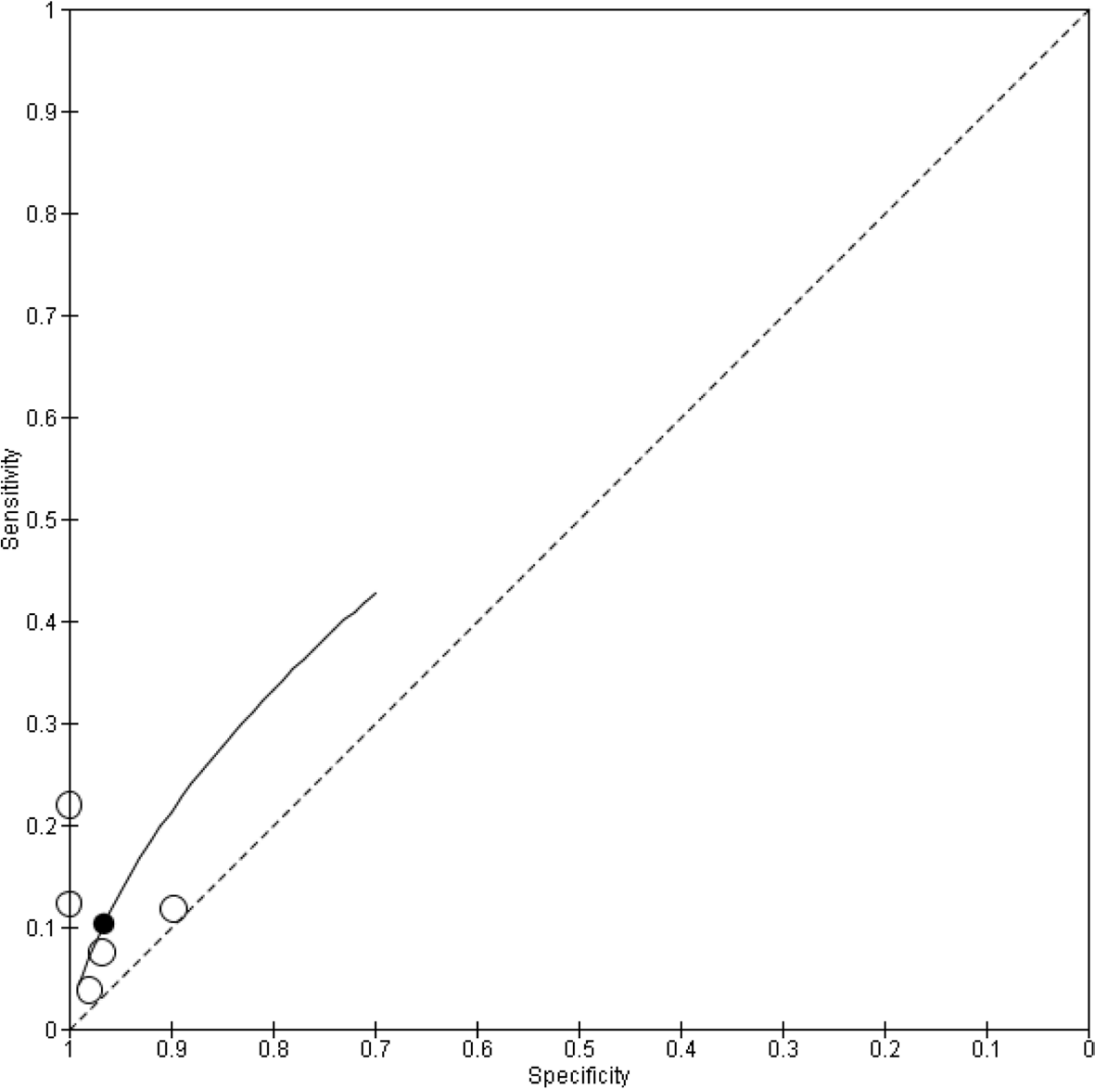
HSROC curve for c-Myc

As observed from the forest plots, there is considerable heterogeneity, which could be attributed to the choice of threshold employed in the studies. In view of this, hierarchical analyses and HSROC curves were used to estimate pooled sensitivity and specificity and these are illustrated in Figs. 4, 5, and 6.

Owing to a small number of studies for hierarchical analysis, summary curves were avoided for HER2, HSP60, Cyclin B1, and P16. However, their diagnostic summary estimates were obtained using random effects model with the help of MetaDTA software. Pooled estimates of individual autoantibodies show a higher specificity as illustrated in Table 2*.* The corresponding diagnostic odds ratio (DOR) show good discriminatory power.

**Table 2:** Pooled estimates of autoantibodies

*Autoantibody*	*Sensitivity (95% CI)*	*Specificity (95% CI)*	*Diagnostic odds ratio (95% CI)*
P53	0.184 (0.093–0.332)	0.962 (0.912–0.984)	5.760 (4.029–8.220)
MUC1	0.498 (0.209–0.789)	0.691 (0.257–0.935)	2.215 (0.440–11.143)
HER2	0.453 (0.064–0.910)	0.794 (0.294–0.973)	3.200 (1.700–6.025)
HSP60	0.558 (0.210–0.857)	0.802 (0.316–0.973)	5.122 (2.145–12.229)
P16	0.216 (0.142–0.314)	0.941 (0.878–0.972)	4.373 (2.329–8.209)
Cyclin B1	0.149 (0.105–0.207)	0.977 (0.946–0.990)	7.455 (2.816–19.733)
c-Myc	0.103 (0.060–0.172)	0.966 (0.919–0.986)	3.230 (1.071–9.740)

### Sensitivity analysis

Sensitivity analysis was carried out with respect to P53, MUC1, and HSP60 as there were differences in certain measures of analysis such as threshold, method, and sample used (Table 3).

**Table 3:** Sensitivity analysis of P53, MUC1, and HSP60 autoantibodies

*Variable*		*Autoantibody*	*Sensitivity (95% CI)*	*Specificity (95% CI)*
Threshold	*Irrespective of the threshold defined (n* = *13)*	P53	0.184 (0.093–0.332)	0.962 (0.912–0.984)
	*Investigator defined-mean* + *2SD (n* = *6)*		0.227 (0.059–0.580)	0.954 (0.794–0.991)
	*Irrespective of the threshold defined (n* = *5)*	MUC1	0.498 (0.209–0.789)	0.691 (0.257–0.935)
	*Investigator defined-mean* + *2SD (n* = *4)*		0.548 (0.196–0.858)	0.615 (0.149–0.936)
Index test	*ELISA* + *immunohistochemistry (n* = *4)*	HSP60	0.558 (0.210–0.857)	0.802 (0.316–0.973)
	*ELISA (n* = *3)*		0.197 (0.097–0.358)	0.979 (0.965–0.988)

In the case of P53 and MUC1, there was a marginal difference in the overall sensitivity measures based on if the threshold used for analysis was investigator defined or manufacturer defined. HSP60 showed higher sensitivity when ELISA was combined with immunohistochemistry. On the other hand, type of sample used for analysis (plasma and serum) did not reflect any difference in overall sensitivity measures between the groups.

## Discussion

### Summary of evidence

Ongoing research in the field of cancer immunology has proposed the utility of serum autoantibodies for early detection of breast cancer [3, 55]. Due to their inherent properties of immune response to tumor-associated antigens, these serum autoantibodies have been investigated for their role as biomarkers in cancer [56, 57].

Few of the most investigated autoantibodies are P53, NY-ESO-1, HER2/neu, MUC1, heat shock proteins, and cyclins [56]. Nevertheless, information is sparse with respect to autoantibodies that are specific to early detection of breast cancer. This was the basis to conduct this systematic review, an initial step in identifying serum autoantibodies as biomarkers for early detection of breast cancer. The aim of this review was to select autoantibodies based on their sensitivity and specificity measures and thus, we planned a DTA review.

Considering its applicability in early detection and hence the need for considerable sensitivity, autoantibody studies have summarily concluded that a panel of autoantibodies demonstrates higher sensitivity than any one individual autoantibody. An example of this is a study by Liu et al. on sera from breast cancer cases and healthy controls wherein a gradual increase in sensitivity was noted from 18.4% for one autoantibody to 67.3% for a panel of six autoantibodies [46]. A similar study by Ye et al. proposed a panel of ten autoantibodies having a sensitivity of 61% in comparison to only 22% for one autoantibody [38]. These findings suggest a clear potential of autoantibody panels in early detection of the disease and thus through this review, we also aimed at selecting a panel of autoantibodies instead of any one individual autoantibody.

This review reports P53, HER2, MUC1, HSP60, P16, Cyclin B1, and c-Myc as the most frequently investigated autoantibodies. A similar review on autoantibodies in breast cancer by Xia et al. reported P53, HER2, MUC1, and Cyclin B1 as autoantibodies with a potential for early detection of breast cancer [56]. Our results further reiterate the role of tumor suppressor and oncogenic proteins such as P53 and c-Myc, respectively in breast cancer. Over-expression of transmembrane mucin proteins such as MUC1 and growth factor like HER2 in breast cancer are sufficiently documented and our results on them support such findings. Of late, Cyclin B1 that is primarily involved in cell-cycle regulation has been investigated for its over- expression in breast and cervix cancers.

A review by Tang et al. who focused on autoantibody signatures in cancer, highlighted c-Myc, P53, NY-ESO-1. HER2, MUC1, and annexin-1 among other autoantibodies as those investigated for early detection of lung cancer [58]. Such findings on autoantibodies in different types of cancer necessitates focused analytical study designs to ascertain autoantibodies that are specific to a particular type of cancer.

We observed that pooled diagnostic odds ratio (DOR) of all the autoantibodies was greater than one implying good discriminating capacity of these autoantibodies. However, the corresponding beta parameter derived from the HSROC model was not equal to zero, suggesting high heterogeneity and therefore the DOR values must be interpreted with caution. Autoantibodies that were most frequently investigated and showed relatively higher pooled sensitivity values namely, HSP60 (55.8%), HER2 (45.3%), MUC1 (49.8%), and P53 (18.4%) were considered for inclusion into a panel for further validation.

### Limitations

Based on the applicability of the review question, we tailored QUADAS-2. On assessment, we observed that most studies were unclear in the domains of ‘reference standard’ and ‘flow and timing’. Although the applicability concern in these domains was ‘low’, this brings to light the shortcomings in reporting of diagnostic studies. This can be attributed to the observation that QUADAS-2 has been designed for diagnostic studies that are cross-sectional in nature. However, the studies in this review have followed a ‘case–control’ design which report elevated values of diagnostic measures.

As heterogeneity is the norm in DTA reviews, DTA experts recommend the use of hierarchical models for meta-analysis and similar other papers on autoantibodies for early detection have adopted this based on their respective review queries [58–61]. Although we used MetaDisc for analysis, considering certain issues with its ongoing software update, we also used the recently developed online application, MetaDTA [10].

## Conclusion

Findings from the review indicate a higher sensitivity for an autoantibody panel in comparison to individual assays. Based on the higher pooled sensitivity values *P53, MUC1, HER2*, and* HSP60* have the potential to be investigated as an early detection biomarker panel for breast cancer. However, as the findings of this review resulted from a heterogenous group of studies especially in terms of population and patient groups, their generalizability for clinical application must be exercised with caution.

## Supplementary Information

**Additional file 1: Table S1.** Search terms and search strategies.

**Additional file 2. **Characteristics of excluded studies.

**Additional file 3. **Data extraction.

**Additional file 4. **QUADAS-2 tool: Risk of bias and applicability judgments.

**Additional file 5: Table S2.** Characteristics of studies with respect to autoantibodies, methods of analysis and population characteristics. **Table S3.** Characteristics of studies with respect to index tests, reference tests, definition of threshold and diagnostic measures

**Additional file 6. **Autoantibodies.

**Additional file 7. **Threshold and methods of analyses.

**Additional file 8. **Study and 2 × 2 tables.

**Additional file 9. **Summary ROC (SROC) curves of autoantibodies.

**Additional file 10.**

## Data Availability

The datasets used and/or analyzed during the current study are available from the corresponding author on request.

## Abbreviations

DTA: Diagnostic Test Accuracy
PRISMA-P: Preferred Reporting Items for Systematic review and Meta-Analysis Protocols
MeSH: Medical Subject Headings
ELISA: Enzyme-linked immunosorbent assay
2DE: Two-dimension electrophoresis
WB: Western blot
MS: Mass spectrophotometry
QUADAS-2: Quality Assessment of Diagnostic Accuracy Studies–2
ROC curve: Receiver operating characteristic curve
AUC: Area under the ROC curve
SROC: Summary ROC
HSROC: Hierarchical summary operating characteristic
SD: Standard deviation
DOR: Diagnostic odds ratio

## References

[1] Sung H, Ferlay J, Siegel RL, Laversanne M., Soerjomataram I, Jemal A. et al. Global Cancer Statistics 2020: GLOBOCAN Estimates of Incidence and Mortality Worldwide for 36 Cancers in 185 Countries. CA Cancer J Clin. 2021. 10.3322/caac.21660 10.3322/caac.21660 33538338

[2] Malvia S, Bagadi SA, Dubey US, Saxena S (2017). Epidemiology of breast cancer in Indian women. Asia Pac J Clin Oncol.

[3] Chapman C, Murray A, Chakrabarti J, Thorpe A, Woolston C, Sahin U (2007). Autoantibodies in breast cancer: their use as an aid to early diagnosis. Ann Oncol.

[4] Pedersen JW, Wandall HH (2011). Autoantibodies as Biomarkers in Cancer. Lab Med.

[5] Desmetz C, Bascoul-Mollevi C, Rochaix P, Lamy P-J, Kramar A, Rouanet P (2009). Identification of a new panel of serum autoantibodies associated with the presence of in situ carcinoma of the breast in younger women. Clin Cancer Res.

[6] Moher D, Shamseer L, Clarke M, Ghersi D, Liberati A, Petticrew M (2015). Preferred reporting items for systematic review and meta-analysis protocols (PRISMA-P) 2015 statement statement. Syst Rev.

[7] Leeflang MMG (2014). Systematic reviews and meta-analyses of diagnostic test accuracy. Clin Microbiol Infect.

[8] Leeflang MMG, Deeks JJ, Gatsonis C, Bossuyt PMM, Cochrane Diagnostic Test Accuracy Working Group. Systematic reviews of diagnostic test accuracy. Ann Intern Med. 2008. 10.7326/0003-4819-149-12-200812160-00008. 10.7326/0003-4819-149-12-200812160-00008 PMC2956514 19075208

[9] Whiting PF, Rutjes AWS, Westwood ME, Mallett S, Deeks JJ, Reitsma JB (2011). QUADAS-2: A Revised Tool for the Quality Assessment of Diagnostic Accuracy Studies. Ann Intern Med.

[10] Review Manager (RevMan) [Computer program]. Version 5.3. Copenhagen: The Nordic Cochrane Centre, The Cochrane Collaboration, 2014.

[11] Freeman SC, Kerby CR, Patel A, Cooper NJ, Quinn T, Sutton AJ (2019). Development of an interactive web-based tool to conduct and interrogate meta-analysis of diagnostic test accuracy studies: MetaDTA. BMC Med Res Methodol.

[12] Chen X, Dong K, Long M, Lin F, Wang X, Wei J (2012). Serum anti-AEG-1 auto-antibody is a potential novel biomarker for malignant tumors. Oncol Lett.

[13] Tomkiel JE, Alansari H, Tang N, Virgin JB, Yang X, VandeVord P (2002). Autoimmunity to the M(r) 32,000 subunit of replication protein A in breast cancer. Clin Cancer Res.

[14] Wu M, Mao C, Chen Q, Cu XW, Zhang WS (2010). Serum p53 protein and anti-p53 antibodies are associated with increased cancer risk: a case-control study of 569 patients and 879 healthy controls. Mol Biol Rep.

[15] Angelopoulou K, Diamandis EP, Sutherland DJA, Kellen JA, Bunting PS (1994). Prevalence of serum antibodies against the p53 tumor suppressor gene protein in various cancers. Int J Cancer.

[16] Nesterova MV, Johnson N, Cheadle C, Bates SE, Mani S, Stratakis CA (2006). Autoantibody cancer biomarker: extracellular protein kinase A. Cancer Res.

[17] Nunna V, Banerjee S, Kumar MK (2014). Circulatory autoantibodies against hyaluronic acid binding proteins: a novel serum biomarker. Asian J Pharm Clin Res.

[18] Wandall HH, Blixt O, Tarp MA, Pedersen JW, Bennett EP, Mandel U (2010). Cancer biomarkers defined by autoantibody signatures to aberrant O-glycopeptide epitopes. Cancer Res.

[19] Regele S, Kohlberger P, Vogl FD, Böhm W, Kreienberg R, Runnebaum IB (1999). Serum p53 autoantibodies in patients with minimal lesions of ductal carcinoma in situ of the breast. Br J Cancer.

[20] Bassaro L, Russell SJ, Pastwa E, Somiari SA, Somiari RI (2017). Screening for multiple autoantibodies in plasma of patients with breast cancer. Cancer Genomics Proteomics.

[21] Chen C, Huang Y, Zhang C, Liu T, Zheng H, Wan S (2015). Circulating antibodies to p16 protein-derived peptides in breast cancer. Mol Clin Oncol.

[22] Evans RL, Pottala JV, Egland KA (2014). Classifying patients for breast cancer by detection of autoantibodies against a panel of conformation-carrying antigens. Cancer Prev Res (Phila).

[23] Liu T, Song Y, Shi Q-Y, Liu Y, Bai X, Pang D (2014). Study of circulating antibodies against CD25 and FOXP3 in breast cancer. Tumour Biol.

[24] Mohammed M, Abdelhafiz K (2015). Autoantibodies in the sera of breast cancer patients: antinuclear and anti-double stranded DNA antibodies as example. J Cancer Res Ther.

[25] Wang J, Figueroa JD, Wallstrom G, Barker K, Park JG, Demirkan G (2015). Plasma Autoantibodies Associated with Basal-like Breast Cancers. Cancer Epidemiol Biomarkers Prev.

[26] Yahalom G, Weiss D, Novikov I, Bevers TB, Radvanyi LG, Liu M (2013). An Antibody-based Blood Test Utilizing a Panel of Biomarkers as a New Method for Improved Breast Cancer Diagnosis. Biomark Cancer.

[27] Yi JK, Chang JW, Han W, Lee JW, Ko E, Kim DH (2009). Autoantibody to tumor antigen, alpha 2-HS glycoprotein: a novel biomarker of breast cancer screening and diagnosis. Cancer Epidemiol Biomarkers Prev.

[28] Balogh GA, Mailo DA, Corte MM, Roncoroni P, Nardi H, Vincent E (2006). Mutant p53 protein in serum could be used as a molecular marker in human breast cancer. Int J Oncol.

[29] Balogh GA, Mailo D, Nardi H, Corte MM, Vincent E, Barutta E (2010). Serological levels of mutated p53 protein are highly detected at early stages in breast cancer patients. Exp Ther Med.

[30] Gao RJ, Bao HZ, Yang Q, Cong Q, Song JN, Wang L (2005). The presence of serum anti-p53 antibodies from patients with invasive ductal carcinoma of breast: correlation to other clinical and biological parameters. Breast Cancer Res Treat.

[31] Kulić A, Sirotković-Skerlev M, Jelisavac-Cosić S, Herceg D, Kovac Z, Vrbanec D (2010). Anti-p53 antibodies in serum: relationship to tumor biology and prognosis of breast cancer patients. Med Oncol..

[32] Lacombe J, Mangé A, Bougnoux A-C, Prassas I, Solassol J (2014). A multiparametric serum marker panel as a complementary test to mammography for the diagnosis of node-negative early-stage breast cancer and DCIS in young women. Cancer Epidemiol Biomarkers Prev.

[33] Lenner P, Wiklund F, Emdin SO, Arnerlöv C, Eklund C, Hallmans G (1999). Serum antibodies against p53 in relation to cancer risk and prognosis in breast cancer: a population-based epidemiological study. Br J Cancer.

[34] Liu Y, Liao Y, Xiang L, Jiang K, Li S, Huangfu M (2017). A panel of autoantibodies as potential early diagnostic serum biomarkers in patients with breast cancer. Int J Clin Oncol.

[35] Lu H, Ladd J, Feng Z, Wu M, Goodell V, Pitteri SJ (2012). Evaluation of known oncoantibodies, HER2, p53, and cyclin B1, in prediagnostic breast cancer sera. Cancer Prev Res.

[36] Pagaza-Straffon C, Marchat LA, Herrera L, Díaz-Chávez J, Avante MG, Rodríguez YP (2020). Evaluation of a panel of tumor-associated antigens in breast cancer. Cancer Biomark.

[37] Qiu C, Wang P, Wang B, Shi J, Wang X, Li T (2020). Establishment and validation of an immunodiagnostic model for prediction of breast cancer. Oncoimmunology.

[38] Ye H, Sun C, Ren P, Dai L, Peng B, Wang K (2013). Mini-array of multiple tumor-associated antigens (TAAs) in the immunodiagnosis of breast cancer. Oncol Lett.

[39] Blixt O, Bueti D, Burford B, Allen D, Julien S, Hollingsworth M (2011). Autoantibodies to aberrantly glycosylated MUC1 in early stage breast cancer are associated with a better prognosis. Breast Cancer Res.

[40] Croce V, Isla-larrain MT, Demichelis SO, Gori JR, Price R, Segal-eiras A (2003). Tissue and serum MUC1 mucin detection in breast cancer patients. Breast Cancer Res Treat.

[41] Tang Y, Wang L, Zhang P, Wei H, Gao R, Liu X (2010). Detection of circulating anti-mucin 1 (MUC1) antibodies in breast tumor patients by indirect enzyme-linked immunosorbent assay using a recombinant MUC1 protein containing six tandem repeats and expressed in Escherichia coli. Clin Vaccine Immunol.

[42] Zuo X, Chen L, Liu L, Zhang Z, Zhang X, Yu Q (2016). Identification of a panel of complex autoantigens (LGALS3, PHB2, MUC1, and GK2) in combination with CA15-3 for the diagnosis of early-stage breast cancer. Tumor Biol.

[43] Desmetz C, Bibeau F, Boissière F, Bellet V, Rouanet P, Maudelonde T (2008). Proteomics-based identification of HSP60 as a tumor-associated antigen in early stage breast cancer and ductal carcinoma in situ. J Proteome Res.

[44] Hamrita B, Chahed K, Kabbage M, Guillier CL, Trimeche M, Chaïeb A (2008). Identification of tumor antigens that elicit a humoral immune response in breast cancer patients’ sera by serological proteome analysis (SERPA). Clin Chim Acta.

[45] Huang Y, Zhang C, Chen C, Sun S, Zheng H, Wan S (2015). Investigation of circulating antibodies to ANXA1 in breast cancer. Tumor Biol.

[46] Liu W, De La Torre IG, Gutiérrez-Rivera MC, Wang B, Liu Y, Dai L (2015). Detection of autoantibodies to multiple tumor-associated antigens (TAAs) in the immunodiagnosis of breast cancer. Tumor Biol.

[47] Liu W, Li Y, Wang B, Dai L, Qian W, Zhang JY (2015). Autoimmune Response to IGF2 mRNA-Binding Protein 2 (IMP2/p62) in Breast Cancer. Scand J Immunol.

[48] Oaxaca-Camacho AR, Ochoa-Mojica OR, Aguilar-Lemarroy A, Jave-Suárez LF, Muñoz-Valle JF, Padilla-Camberos E (2020). Serum analysis of women with early-stage breast cancer using a mini-array of tumor-associated antigens. Biosensors.

[49] Dong X, Yang M, Sun H, Lü J, Zheng Z, Li Z (2013). Combined measurement of CA 15–3 with novel autoantibodies improves diagnostic accuracy for breast cancer. Onco Targets Ther.

[50] Fernández-Grijalva AL, Aguilar-Lemarroy A, Jave-Suarez LF, Gutiérrez-Ortega A, Godinez-Melgoza PA, Herrera-Rodríguez SE (2015). Alpha 2HS-glycoprotein, a tumor-associated antigen (TAA) detected in Mexican patients with early-stage breast cancer. J Proteomics.

[51] Frenkel K, Karkoszka J, Glassman T, Dubin N, Toniolo P, Taioli E (1998). Serum autoantibodies recognizing 5-hydroxymethyl-2 ’ -deoxyuridine, an oxidized DNA base, as biomarkers of cancer risk in women. Cancer Epidemiol Biomarkers Prev.

[52] Le Naour F, Misek DE, Krause MC, Deneux L, Giordano TJ, Scholl S (2001). Proteomics-based identification of RS/DJ-1 as a novel circulating tumor antigen in breast cancer. Clin Cancer Res.

[53] Kurtenkov O, Innos K, Sergejev B, Klaamas K (2018). The thomsen-friedenreich antigen-specific antibody signatures in patients with breast cancer. Biomed Res Int.

[54] Zhong L, Ge K, Zu JC, Zhao LH, Shen WK, Wang JF (2008). Autoantibodies as potential biomarkers for breast cancer. Ovid Medlin Cancer Res.

[55] Piura E, Piura B (2011). Autoantibodies to tailor-made panels of tumor-associated antigens in breast carcinoma. J Oncol.

[56] Dudas SP, Chatterjee M, Tainsky MA (2010). Usage of cancer associated autoantibodies in the detection of disease. Cancer Biomark.

[57] Macdonald IK, Parsy-Kowalska CB, Chapman CJ (2017). Autoantibodies: Opportunities for Early Cancer Detection. Trends in Cancer.

[58] Tang ZM, Ling ZG, Wang CM, Kong JL, Wu Y Bin (2017). Serum tumor-associated autoantibodies as diagnostic biomarkers for lung cancer: a systematic review and meta-analysis. PLoS One..

[59] Xia J, Shi J, Wang P, Song C, Wang K, Zhang J (2016). Tumour-associated autoantibodies as diagnostic biomarkers for breast cancer: a systematic review and meta-analysis. Scand J Immunol.

[60] Werner S, Chen H, Tao S, Brenner H (2015). Systematic review: Serum autoantibodies in the early detection of gastric cancer. Int J Cancer.

[61] Zhang H, Xia J, Wang K, Zhang J (2014). Serum autoantibodies in the early detection of esophageal cancer: a systematic review. Tumor Biol.

