# Supplementary material for: Can serum autoantibodies be a potential early detection biomarker for breast cancer in women? A diagnostic test accuracy review and meta-analysis
Source: Syst Rev. 2022 Oct 9;11:215. doi: 10.1186/s13643-022-02088-y (PMC9549667; doi:10.1186/s13643-022-02088-y)
Supplement: Supplementary file 1 — Additional file 1: Table S1. Search terms and search strategies. [file 13643_2022_2088_MOESM1_ESM.docx]

| Table S1: Search terms and search strategies | | | | | |
| --- | --- | --- | --- | --- | --- |
| Database | MEDLINE *^a^ | Scopus *^b^ | ProQuest *^c^ | Ovid SP *^d^ | Cochrane library *^e^ |
| Search set |  |  |  |  |  |
| 1 | breast | breast | breast | breast | breast |
| 2 | “breast cancer” | “breast cancer” | “breast cancer” | “breast cancer” | “breast cancer” |
| 3 | “BREAST CARCINOMA” | “breast carcinoma” | “BREAST CARCINOMA” | “breast carcinoma” | “breast carcinoma” |
| 4 | 1 or 2 or 3 | 1 or 2 or 3 | 1 or 2 or 3 | 1 or 2 or 3 | 1 or 2 or 3 |
| 5 | “TUMOUR ^#^ ASSOCIATED ANTIGENS” | “tumour associated antigens” | “TUMOUR ASSOCIATED ANTIGENS” | “tumour associated antigens” | “tumour associated antigens” |
| 6 | antibodies | antibodies | antibodies | antibodies | antibodies |
| 7 | AUTOANTIBODIES | autoantibodies | AUTOANTIBODIES | autoantibodies | autoantibodies |
| 8 | 5 or 6 or 7 | 5 or 6 or 7 | 5 or 6 or 7 | 5 or 6 or 7 | 5 or 6 or 7 |
| 9 | serum | serum | serum | serum | serum |
| 10 | “biomarker” | “biomarker” | “biomarker” | “biomarker” | “biomarker” |
| 11 | blood | blood | blood | blood | blood |
| 12 | 9 or 10 or 11 | 9 or 10 or 11 | 9 or 10 or 11 | 9 or 10 or 11 | 9 or 10 or 11 |
| 13 | SCREENING | screening | screening | screening | screening |
| 14 | detection | detection | detection | detection | detection |
| 15 | “early detection” | “early detection” | “early detection” | “early detection” | “early detection” |
| 16 | 13 or 14 or 15 | 13 or 14 or 15 | 13 or 14 or 15 | 13 or 14 or 15 | 13 or 14 or 15 |
| 17 | 4 and 8 and 12 and 16 | 4 and 8 and 12 and 16 | 4 and 8 and 12 and 16 | 4 and 8 and 12 and 16 | 4 and 8 and 12 and 16 |

^*^ search terms combined and search executed as per respective database styles; lower case – free text term

^a^ upper case – MESH headings; ^#^ word variation searched; limit to human

^b^ limit to title and abstract

^c^ upper case – MESH headings; limit to title, abstract

^d^ limit to title, abstract

^e^ limit to title, abstract, keyword
