# Supplementary material for: Can serum autoantibodies be a potential early detection biomarker for breast cancer in women? A diagnostic test accuracy review and meta-analysis
Source: Syst Rev. 2022 Oct 9;11:215. doi: 10.1186/s13643-022-02088-y (PMC9549667; doi:10.1186/s13643-022-02088-y)
Supplement: Supplementary file 2 — Additional file 2. Characteristics of excluded studies. [file 13643_2022_2088_MOESM2_ESM.docx]

Characteristics of excluded studies

| Studies not meeting the inclusion criteria | 66 |
| --- | --- |
| Book chapter | 2 |
| Narrative reviews | 36 |
| *Total* | *104* |
