# Supplementary material for: Can serum autoantibodies be a potential early detection biomarker for breast cancer in women? A diagnostic test accuracy review and meta-analysis
Source: Syst Rev. 2022 Oct 9;11:215. doi: 10.1186/s13643-022-02088-y (PMC9549667; doi:10.1186/s13643-022-02088-y)
Supplement: Supplementary file 3 — Additional file 3. Data extraction. [file 13643_2022_2088_MOESM3_ESM.docx]

|  | | | |
| --- | --- | --- | --- |
| 1. **STUDY DESCRIPTION** | | | |
| 1. Study | |  | |
| 1. Author and Year | |  | |
| 1. Country | |  | |
| 1. Funding source | |  | |
| 1. **CHARACTERISTICS OF THE STUDY POPULATION** | | | |
| 1. Study design | | |  |
| 1. Prospective / retrospective | | |  |
| 1. Recruitment method | | |  |
| 1. Multicentre | | |  |
| 1. Enrolment years | | |  |
| 1. Number of breast cancer patients | | |  |
| 1. Number of healthy individuals | | |  |
| 1. Age   Cases | | |  |
| Controls | | |  |
| 1. Cases | | |  |
| Exclusion criteria | | |  |
| 1. Controls | | |  |
| Exclusion criteria | | |  |
| 1. Matching; confounding factors | | |  |
| 1. **DISEASE CHARACTERISTICS AND SAMPLE ANALYSIS** | | | |
| 1. Disease stage | | |  |
| 1. Number of cases with each disease stage | | |  |
| 1. **METHODOLOGY** | | | |
| **SAMPLE ANALYSIS** | | | |
| 1. Type of sample used for analysis | | |  |
| 1. Type of kit(s) used –   Commercially available / in-  house preparation | | |  |
| 1. Method(s) of analysis | | |  |
| **REFERENCE TEST AND INDEX TEST** | | |  |
| 1. Description of the reference test/comparator test | | |  |
| 1. Time of testing ( before or after the index test ) | | |  |
|  | | |  |
| 1. Description of the index test | | |  |
| 1. Time of testing (before or after the reference test) | | |  |
|  | | |  |
| Were the results of reference test blinded to that of the index test? | | |  |
| **AUTOANTIBODIES** | | |  |
| 1. Autoantibodies tested | | |  |
| 1. Type of assay :   Single antibody / panel / both | | |  |
| 1. Significant autoantibodies | | |  |
| 1. Cut –off value for positive reactivity | | |  |
| 1. **STATISTICAL METHODS** | | | |
| 1. **RESULTS** | | | |
|  | | | |
| 1. Sensitivity |  | | |
| 1. Specificity |  | | |
| 1. Reproducibility of the assay |  | | |
| 1. Positive predictive value (PPV) |  | | |
| 1. Negative predictive value (NPV) |  | | |
| 1. ROC analysis |  | | |
| 1. **FINAL CONCLUSIONS OF THE AUTHORS** | | | |
| 1. **QUALITY ASSESSMENT**   **Domain 1: Patient selection**  A. Risk of Bias   1. Was a consecutive or random sample of patients enrolled? Yes/No/Unclear 2. Was a case-control design avoided? Yes/No/Unclear 3. Did the study avoid inappropriate exclusions? Yes/No/Unclear   Could the selection of patients have introduced bias?  RISK: LOW/HIGH/UNCLEAR  B. Concerns regarding applicability  Is there concern that the included patients do not match the review question?  CONCERN: LOW/HIGH/UNCLEAR  **Domain 2: Index test(s)**  A. Risk of Bias   1. Were the index test results interpreted without the knowledge Yes/No/Unclear   of the results of the reference standard?   1. If a threshold was used, was it pre-specified? Yes/No/Unclear   Could the conduct or interpretation of the index test have introduced bias?    RISK: LOW /HIGH/UNCLEAR  B. Concerns regarding applicability  Is there concern that the index test, its conduct, or interpretation differ from the review question?  CONCERN: LOW /HIGH/UNCLEAR  **Domain 3: Reference standard**  A. RISK OF BIAS   1. Is the reference standard/ comparator test likely to correctly classify the target Yes/No/Unclear   condition?  Could the reference standard, its conduct, or its interpretation have introduced bias?  RISK: LOW /HIGH/UNCLEAR  B. Concerns regarding applicability  Is there concern that the target condition as defined by the reference standard does not match the review question?  CONCERN: LOW /HIGH/UNCLEAR  **Domain 4: Flow and timing**  A. RISK OF BIAS   1. Did all patients receive a reference standard/ comparator test? Yes/No/Unclear 2. Did patients receive the same reference standard/ comparator test? Yes/No/Unclear 3. Were all patients included in the analysis? Yes/No/Unclear   Could the patient flow have introduced bias?  RISK: LOW /HIGH/UNCLEAR | | | |
