# Supplementary material for: Can serum autoantibodies be a potential early detection biomarker for breast cancer in women? A diagnostic test accuracy review and meta-analysis
Source: Syst Rev. 2022 Oct 9;11:215. doi: 10.1186/s13643-022-02088-y (PMC9549667; doi:10.1186/s13643-022-02088-y)
Supplement: Supplementary file 4 — Additional file 4. QUADAS-2 tool: Risk of bias and applicability judgments. [file 13643_2022_2088_MOESM4_ESM.doc]

QUADAS-2 tool: Risk of bias and applicability judgments

| **Domain 1: Patient selection** | |
| --- | --- |
| 1. Risk of bias |  |
| Describe methods of patient selection: | |
| - Was a consecutive or random sample of patients enrolled? | Yes/No/Unclear |
| - Was a case-control design avoided? | Yes/No/Unclear |
| - Did the study avoid inappropriate exclusions? | Yes/No/Unclear |
| Could the selection of patients have introduced bias? | RISK: LOW/HIGH/UNCLEAR |
| 1. Concerns regarding applicability |  |
| Describe included patients (prior testing, presentation, intended use of index test and setting): | |
| Is there concern that the included patients do not match the review question? | CONCERN: LOW/HIGH/UNCLEAR |
| **Domain 2: Index test(s) *(if more than 1 index test was used, please complete for each test)*** | |
| 1. Risk of bias |  |
| Describe the index test and how it was conducted and interpreted: | |
| - Were the index test results interpreted without knowledge of the results of the reference standard? | Yes/No/Unclear |
| - If a threshold was used, was it pre-specified? | Yes/No/Unclear |
| Could the conduct or interpretation of the index test have introduced bias? | RISK: LOW/HIGH/UNCLEAR |
| 1. Concerns regarding applicability |  |
| Is there concern that the index test, its conduct, or interpretation differ from the review question? | CONCERN: LOW/HIGH/UNCLEAR |
| **Domain 3: Reference standard** | |
| 1. Risk of bias |  |
| Describe the reference standard and how it was conducted and interpreted: | |
| - Is the reference standard/ comparator test likely to correctly classify the target condiiton? | Yes/No/Unclear |
| Could the reference standard, its conduct, or its interpretation have introduced bias? | RISK: LOW/HIGH/UNCLEAR |
| 1. Concerns regarding applicability |  |
| Is there concern that the target condition as defined by the reference standard/ comparator test does not match the review question? | CONCERN: LOW/HIGH/UNCLEAR |
| **Domain 4: Flow and timing** | |
| 1. Risk of bias |  |
| Describe any patients who did not receive the index test(s) and/or reference standard or who were excluded from the 2x2 table (refer to flow diagram):  Describe the time interval and any interventions between index test(s) and reference standard: | |
| - Did all patients receive a reference/ comparator test standard? | Yes/No/Unclear |
| - Did all participants receive the same reference standard/ comparator test? | Yes/No/Unclear |
| - Were all patients included in the analysis? | Yes/No/Unclear |
| Could the patient flow have introduced bias? | RISK: LOW/HIGH/UNCLEAR |
