# Supplementary material for: Can serum autoantibodies be a potential early detection biomarker for breast cancer in women? A diagnostic test accuracy review and meta-analysis
Source: Syst Rev. 2022 Oct 9;11:215. doi: 10.1186/s13643-022-02088-y (PMC9549667; doi:10.1186/s13643-022-02088-y)
Supplement: Supplementary file 5 — Additional file 5: Table S2. Characteristics of studies with respect to autoantibodies, methods of analysis and population characteristics. Table S3. Characteristics of studies with respect to index tests, reference tests, definition of threshold and diagnostic measures [file 13643_2022_2088_MOESM5_ESM.docx]

| *Table S2: Characteristics of studies with respect to autoantibodies, methods of analysis and population characteristics* | | | | | | |
| --- | --- | --- | --- | --- | --- | --- |
| Sl No | Author, Country & year | Autoantibody investigated | Type of sample for analysis | Technique | Study design | Cases, controls/ patients, healthy |
|  | Anderson K S et al., 2011[62] | ATP6AP1 | Sera | NAPPA (Novel high-density custom protein microarray) and ELISA | Reversed flow design (case- control) | Patients:  1.Test set - 53  2. Training set - 51  3. Validation set - 51  4. Independent set – 148  Healthy:  1.Test set - 53  2. Training set - 39  3. Validation set - 38  4. Independent set – 64 |
|  | Angelopoulou et al.,  Canada, 1994 [15] | P53 | Sera | 2- time resolved immunofluorometric technique | Reversed flow design (case- control) *(no information whether sera were collected before after treatment)* | Patients: 290 (along with other types of cancers)  Healthy: 230 (included hospitalized patients and individuals with co-morbid illness) |
|  | Balogh et al., 2005, Argentina [28] | P53 | Sera and tissue | ELISA, immunohistochemistry, RT-PCR | Reversed flow design (case- control) | Patients: 55  Healthy: 8 |
|  | Balogh et al., 2009,  Argentina [29] | P53 | Sera | ELISA, immunohistochemistry | Reversed flow design (case- control) | Patients: 55  Healthy: 8 |
|  | Bassaro et al., 2017, USA [20] | interleukin 29 (IL29), osteoprotegerin (OPG), survivin (SUR), growth hormone (GRH) and resistin (RES) | Plasma | APS (antigen profiling system)/ ELISA | Reversed flow design (case- control) | Patients: 9  Healthy: 9 |
|  | Ola Blixt et al., 2011, London [39] | MUC1 | Sera | Microarray technique | Reversed flow design (case- control) | Patients: 395  Healthy: 99 |
|  | Camacho et al., 2020, Mexico [48] | Alpha 1-AntiTrypsin (A1AT), TriosePhosphate Isomerase 1 (TPI1), Peptidyl-Prolyl cis-trans isomerase A (PPIA) and PeroxiReDoXin 2 (PRDX2) | Sera | Western blot, dot- blot | Reversed flow design (case- control) | Patients: 12  Healthy: 12 |
|  | Chapman et al., 2007,  UK, Germany [3] | P53, c-Myc, HER2, NY-ESO-1, BRCA1, BRCA2 and MUC1  (panel and single antibody) | Sera | ELISA | Reversed flow design (case- control) | Patients: 137  Healthy: 94 |
|  | Chen C et al., 2014, China [21] | p16 | Plasma | ELISA | Reversed flow design (case- control) | Patients: 152  Healthy: 160 |
|  | Chen X et al., 2012, China [12] | Astrocyte elevated gene – 1 (AEG -1) | Sera | ELISA | Reversed flow design (case- control) | Patients: 98  Healthy: 115 (included other cancers and males) |
|  | Nesterova et al., 2006, USA [16] | ECPKA (extracellular protein kinase A) | Sera | EIA (ELISA) | Reversed flow design (case- control) | Patients: 24  Healthy: 155  (included other cancers and males) |
|  | Croce et al., 2003, Argentina [40] | MUC1 | Sera and tissue | ELISA | Reversed flow design (case- control) | Patients: 70  Healthy: 135  (healthy population consisted of post and pre vaccination samples) |
|  | Desmetz et al., 2008, France [43] | HSP60 | Sera, tissue and cell lines | 2 DE Western Blot analysis,  IHC, ELISA | Reversed flow design (case- control) | Patients: 107 (for ELISA) Healthy: 93 |
|  | Dong et al., 2013, China [49] | CA-15.3 antigen, hnRNPF and FTH1 abs | Sera | Biopanning, ELISA | Reversed flow design (case- control) | Patients: 155  Healthy: 155 |
|  | Desmetz et al., 2009, France [5] | PPIA, PRDX2, FKBP52, HSP60 and MUC1 | Sera and tissue | ELISA, Western Blot and proteomics | Reversed flow design (case- control) | Patients: 142  Healthy: 93 |
|  | Evans et al., 2014, USA [22] | ANGPTL4, DKK1, EPHA2, GAL1, HER-2, IGFBP2, LAMC2, MUC1, SPON2, CST2, SPINT2 and SSR2. | Plasma | Conformation – carrying antigen ELISA | Reversed flow design (case- control) | Patients: 200  Healthy: 200 |
|  | Fernandez Madrid et al., 2004 [63] | Annexin XI – A | Sera and tissue | Microarray and histological testing | Reversed flow design (case- control) | Patients: 90  Training set – 45  Independent set – 45  Healthy: 51  Training set -26  Independent set - 25 |
|  | Fernandez Grijalva et al., 2014, Mexico [50] | Alpha 2HS glycoprotein | Sera and tissue | Two-dimensional (2D) electrophoresis, Western blot, and matrix-associated laser desorption/ionization-mass spectrometry (MALDI-MS), IHC | Reversed flow design (case- control) | Patients: 36  Healthy: 36 |
|  | Frenkel K et al., 1998  New York [51] | Anti- HMdU | Sera | ELISA | Case- control study | Patients: 9  Healthy: 48 |
|  | Gao et al., 2005, China [30] | P53 | sera and tissue | ELISA, IHC | Reversed flow design (case- control) | Patients: 144  Healthy: 242 |
|  | Hamrita et al., 2008, Tunisia [44] | HSP60, alpha B crystallin, hnRNPK, hnRNPH3, beta tubulin, prohibitin, Mn-SOD, PD1 | Sera and tissues | 2D immunoblot, IHC | Reversed flow design (case- control) | Patients: 40  Healthy: 42 |
|  | Huang et al.,2015, China [45] | ANXA1 | Sera | ELISA | Reversed flow design (case- control) | Patients: 152  Healthy: 160 |
|  | Kyo Yi et al., 2009, Korea [27] | Alpha 2 –HS glycoprotein | Sera (Urine) | Immunoblotting, SDS PAGE/ Western blotting | Reversed flow design (case- control) | Patients: 81  Healthy: 73 |
|  | Kulic et al., 2009, Croatia [31] | P53 | Sera | ELISA | Reversed flow design (case- control) | Patients: 61  Healthy: 20 |
|  | Lacombe et al., 2013, France [64] | GAL 3, PAK 2, PHB2, RACK1and RUVBL1 | Sera | ELISA and proteomic approach | Reversed flow design (case- control) | Patients:  Discovery population – 20  Validation set – 104  Healthy:  Discovery population – 60  Validation set – 68 |
|  | Lacombe et al., 2014, France [32] | HSP60, FKBP52, PRDX2, PP1A, MUC1, GAL3, PAK2, P53, CCNB1, PHB2, RACK1, RUVBL1 and HER2 | Sera | ELISA | Reversed flow design (case- control) | Patients: 240  Healthy: 156 |
|  | Naour et al., 2001, France [52] | RS/ DJ – 1 | Sera and tissue | 2D, Western blot, IHC | Reversed flow design (case- control) | Patients: 30  Healthy: 25 |
|  | Lenner et al., 1999, Sweden [33] | P53 | Sera | ELISA, Western blot | Case- control study | Patients: 165  Healthy: 330 |
|  | Liu, T et al., 2014, China [23] | CD25, FOXP3 | Plasma | ELISA | Reversed flow design (case- control) | Patients: 152  Healthy: 112 |
|  | Liu W et al., 2015, Texas [47] | IMP2/p62 | Sera and tissue | ELISA, Western blot, indirect immunofluorescence | Reversed flow design (case- control) | Patients: 49  Healthy: 44 |
|  | Liu W et al., 2015, Texas [46] | Imp1, p16, Koc, survivin, Cyclin B1, and c-Myc | Sera | ELISA and Western blot | Reversed flow design (case- control) | Patients: 49  Healthy: 38 |
|  | Liu X et al., 2014, USA [65] | p90/CIP2A | Sera and tissue | ELISA, Western Blotting, Indirect Immunofluorescence, IHC. | Reversed flow design (case- control) | Patients: 168  Healthy: 88 |
|  | Liu Y et al., 2017, China [34] | P16, c- Myc, TP53, ANXA-1 | Sera | ELISA | Reversed flow design (case- control) | Patients: 102  Healthy: 146 |
|  | Lopez et al., 2012, Mexico [66] | Alpha 1 antitrypsin | Sera | 2DE, Western Blot, MALDI – MS | Reversed flow design (case- control) | Patients: 25  Healthy: 20 |
|  | Lu H et al., 2012, USA [35] | HER-2/neu, P53, topo 2 alpha, MUC1, CEA, catD and Cyclin B1 | Sera | ELISA | Reversed flow design (case- control) | Patients: 151  Initial triage: 98  Primary validation- 20  Secondary validation – 33  Healthy: 163  Initial triage: 98  Primary validation: 20  Secondary validation: 45 |
|  | Mohammed et al., 2015  Saudi Arabia [24] | Antinuclear and anti - double  stranded DNA antibodies | Plasma | ELISA | Case- control study | Patients: 35  Healthy: 18 |
|  | Nunna et al., 2014, India [17] | Hyaluronic acid binding proteins | Sera | ELISA, Western blot | Reversed flow design (case- control) | Patients: 20  Healthy: 50  (other types of cancers included) |
|  | Oleg et al., 2018, Estonia [47] | Thomsen-Friedenreich Antigen-Specific Antibody and its isotopes | Sera | ELISA | Reversed flow design (case- control) | Patients: 196  Healthy: 64 |
|  | Olga et al., 2017, Russia [67] | RAD50, PARD3, SPP1, SAP30BP, NY-BR-62 and NY-CO-58 | Sera and tissue | ELISA, SEREX, qPCR | Reversed flow design (case- control) | Patients: 112  Healthy: 35 |
|  | Pagaza-Straffon et al.  2020, Mexico [36] | CEA, c-Myc, p53, Ki-67, Nm23, PRDX6, eIF5A, GLIO-1and Hsp70 | Sera | ELISA | Reversed flow design (case- control) | Patients: 104  Healthy: 50 |
|  | Qiu et al., 2019  China [37] | p53, cyclinB1, p16, p62, 14-3-3ξ | Sera | ELISA, Western Blotting | Reversed flow design (case- control) | Validation cohort  Patients: 197  Healthy: 109 |
|  | Regele et al., 1999, Germany [19] | P53 | Sera | ELISA, IHC | Cross- sectional study | 43 |
|  | Tang et al., 2010, China [41] | MUC1 | Sera | ELISA | Reversed flow design (case- control) | Patients: 32  Healthy: 56 (in addition to 90 patients after treatment) |
|  | Tomkiel et al., 2002  Detroit, USA [13] | RPA32 (replication protein A) | Sera | ELISA | Reversed flow design (case- control) | Patients: 801  Healthy: 46 females (the control group included 19 males and had a diagnosis of fibromyalgia and osteoarthritis) |
|  | Wandall et al., 2010, UK [18] | MUC 1 | Sera | Microarray | Reversed flow design (case- control) | Patients:26  Healthy: 33  (also included other cancers) |
|  | Wang J et al., 2015, Poland [25] | CTAG1B, CTAG2, TP53, RNF216, PPHLN1, PIP4K2C, ZBTB16, TAS2R8, WBP2NL, DOK2, PSRC1, MN1, TRIM21 | Plasma | ELISA, NAPPA | Reversed flow design (case- control) | Patients:145  Healthy: 145 |
|  | Wu et al., 2009  China [14] | P53 | Sera | ELISA | Reversed flow design (case- control) | Patients:25  Healthy: 879 |
|  | Yagihashi et al., 2005, Japan [68] | Survivin, livin | Sera | ELISA | Reversed flow design (case- control) | Patients: 46  Healthy: 10 |
|  | Yahalom et al., 2013, USA [26] | 13 antibody marker panel | Plasma | ELISA | Reversed flow design (case- control) | Patients: 201  Healthy: 345 |
|  | Ye et al., 2012  China [38] | c‑myc, survivin, Cyclin B1, cyclin D1, p62, P53, p12 and CDK2, | Sera | ELISA | Reversed flow design (case- control) | Patients: 41  Healthy: 82 |
|  | Zhong et al., 2008, China [54] | KLF 17, COL6A1, GRWD1, ASB-9, SERAC1, and RELT | Sera | ELISA | Reversed flow design (case- control) | Patients: 87  Healthy: 87 |
|  | Zhu et al., 2015, USA [69] | PARP1, BRCA1 and BRCA2 | Sera and tissue | ELISA, Western blot and Immunohistochemistry | Reversed flow design (case- control) | Patients: 131  Healthy: 135 |
|  | Zuo et al., 2014, China [42] | LGALS3, Phb2, MUC1, GK2, CA 15-3 | Sera and tissue | Plaque assay, bio-panning and Western Blotting | Reversed flow design (case- control) | Patients: 110  Healthy: 55 |

| *Table S3: Characteristics of studies with respect to index tests, reference tests, definition of threshold and diagnostic measures* | | | | | | | | | |
| --- | --- | --- | --- | --- | --- | --- | --- | --- | --- |
| Sl. No | Author, Country & year | Reference test/ comparator test | Index test/s | Threshold | Reporting of results | | | | |
|  |  |  |  |  | Sensitivity | Specificity | OR | DOR | Other measures |
|  | Anderson K S et al., 2011[62] | Routine mammography -patient group | NAPPA arrays, ELISA | Threshold was determined by computing the 95% empirical percentile of the normalized  intensity values of the controls. | 1. 28 antigen panel in the combined cohort of 102 cases and 77 controls  44.1% | 96.1% |  | 19.47 |  |
|  |  |  |  |  | 2. ATP6AP1 in an independent set of cohort of 148 cases and 64 controls  12.8% | 95% |  | 3 |  |
|  | Angelopoulou et al.,  Canada, 1994 [15] | ‘diagnosis’ – no mention of the method.  No mention for healthy group | 2- time resolved immunofluorometric technique | Positive samples were identified based on a cut-off fluorescence ratio of 1.7 | 3.4% | 100% |  |  |  |
|  | Balogh et al., 2005, Argentina [28] | Histologic diagnosis - patient group.  No mention for healthy group | ELISA (commercial kit) | The critical range of values was determined as the cut-off ± 20%, means 0.267±20%; the critic range value is 0.214-0.305. The positive values were >0.305 and negative values were <0.214 | 16..36% | 100% |  |  |  |
|  | Balogh et al., 2009,  Argentina [29] | Unclear | ELISA (commercial) | Unclear | 32% (IDC I), 14% (IDC II), 47% (IDC III) |  |  |  |  |
|  | Bassaro et al., 2017, USA [20] | Clinical diagnosis of cancer (DCIS or IDC) | Commercial (Autoantibody profiling system – APS)/ ELISA | OD 450 nm for an autoantibody was =1.5 of the mean of the group, the autoantibody was classified as being elevated in that sample and if <1.5 of the mean OD of the group, that autoantibody was classified as not being elevated in that sample |  |  |  |  | Mean concentration of auto antibody levels compared between cases and controls. |
|  | Ola Blixt et al., 2011, London [39] | Unclear | Microarray | A positive value was defined as being two standard deviations (SDs) above the mean of the reactivity of sera from patients with benign breast disease or healthy female controls, on the corresponding feature. | 31% | 14% |  | 0.07 |  |
|  | Camacho et al., 2020, Mexico [48] | Clinical diagnosis | Western blot, dot- bot | Unclear | TPI1 – 100%, PRDX2 – 87.5%, PPIA – 87.5%, A1AT – 83.3%  Panel – 88.9% | TPI1 – 75%, PRDX2 – 68.8%, PPIA – 68.8%, A1AT – 83.3%  Panel – 73.3% |  |  | *PPV*  TPI1 – 0.7  PRDX2 – 0.6 PPIA – 0.6 A1AT – 0.8  Panel –0.8  *NPV*  TPI1 – 1  PRDX2 – 0.9 PPIA – 0.9  A1AT – 0.8  Panel – 0.9 |
|  | Chapman et al., 2007,  UK, Germany [3] | Unclear | ELISA | An absorbance value greater than the mean +2 standard deviations (SDs) of the normal cohort | *PBC*  P53 – 24%  c-Myc -13%  NY-ESO-1- 26%  BRCA-1 - 8%  BRCA-2 - 34%  HER-2 – 18%  MUC1 – 20%  Panel – 64%  *DCIS*  P53 – 15%  c-Myc -8%  NY-ESO-1- 8%  BRCA-1 - 3%  BRCA-2 - 23%  HER-2 – 13%  MUC1 – 23%  Panel – 45% | P53 – 96%  c-Myc – 97%  NY-ESO-1- 94%  BRCA-1 – 91%  BRCA-2- 92%  HER-2 – 94%  MUC1 – 98%  Panel – 85% |  | PBS  P53 - 7  c-Myc - 4.7  NY-ESO -1 - 5.1  BRCA -1 - 0.91  BRCA-2 – 5.54  HER 2 -3.1  MUC1 -11.2  Panel – 10.12  DCIS  P53 – 3.97  c-Myc - 2.45  NY-ESO -1 – 2.45  BRCA -1 - 0.27  BRCA-2 – 3.12  HER 2 -2.09  MUC1 -3.12  Panel – 4.67 |  |
|  | Chen C et al., 2014, China [21] | Radiographic examination and histological conformation – patient group  Clinical interview and radiographic examination – healthy group | ELISA | Unclear | 30.3% | 90% |  | 6.9 | AUC – 0.74 (95% CI: 0.65 -0.83) |
|  | Chen X et al., 2012, China [12] | Unclear | ELISA | Unclear | 45%  (Given as positive rates from which sensitivity and specificity values were computed by the reviewer) | 100% |  |  |  |
|  | Nesterova et al., 2006, USA [16] | Unclear for patients  Normal sera from blood bank – healthy group | ELISA (EIA) | Antibody titers are arbitrarily expressed as ratios to the mean absorbance of the normal control sera. Values >1.3 were considered positive. | 83% (breast cancer) | 84% |  | 25.85 | AUC- 0.93 (CI -0.8 – 0.95) |
|  | Croce et al., 2003, Argentina [40] | Unclear | ELISA | 0.227 for anti-MUC1 IgG and 0.340 for anti-MUC1 IgM. | IgM – 14%, IgG – 32%  (Given as positive rates from which sensitivity values were computed by the reviewer) |  |  |  |  |
|  | Desmetz et al., 2008, France [43] | Unclear | 2DEWestern blot and ELISA | Absorbance greater than 2SDs above the mean value of the controls | DCIS – 32.6%  Early stage – 31% | 95.7% |  | DCIS – 10.78  Early stage – 10.01 | *Healthy v DCIS*  PPV -89.5%  NPV – 54.9%  Healthy v early stage  PPV – 81.8%  NPV – 70.0% |
|  | Dong et al., 2013, China [24] | Unclear | ELISA | For FTH1 – 0.98 at OD of 450nm  For hnRNPF – 1.09 at OD of 45nnm | FTH1 -81.2%, hnRNPF -84.2%,  CA-15.3 – 69.1  FTH1 + hnRNPF -91.1%,  FTH1+ CA-15.3 –85.1%  hnRNPF + CA-15.3 – 87.4%  All 3 – 89.3% | FTH1 – 56.1%, hnRNPF - 60.8%, CA-15.3 – 89.4%  FTH1 + hnRNPF – 72.0%, FTH1+ CA-15.3 – 92.7% hnRNPF + CA-15.3 – 91.0%  All 3 – 93.8% |  | FTH1 – 5.55  HnRNPF – 7.6  FTH1 + hnRNPF – 26.23  FTH1 + hnRNPF + CA15-3 – 131.7 |  |
|  | Desmetz et al., 2009, France [5] | Unclear | ELISA, Western blot | Unclear | HC/Cancer  FKBP52 – 50.1%  PP1A – 49.6%  PRDX2 – 45.2%  HSP60 – 35%  MUC1 – 36.5% | HC/Cancer  FKBP52 – 87.2%  PP1A – 87.3%  PRDX2 – 85.5%  HSP60 – 87.1%  MUC1 – 87.9% |  | HC/Cancer  FKBP52 – 6.75  PP1A – 6.75  PRDX2 – 2.68  HSP60 – 3.66  MUC1 – 4.43 | HC/Cancer  *PPV*  FKBP52 – 85.7  PP1A – 85.6  PRDX2 – 82.6  HSP60 – 80.5  MUC1 – 82.1  *NPV*  FKBP52 – 5.34  PP1A – 53.2  PRDX2 – 50.6  HSP60 – 46.8  MUC1 – 47.6 |
|  | Evans et al., 2014, USA [22] | Unclear – patient group  Mammogram - healthy group | ELISA | Absorbance greater than 2SDs above the mean value of the controls. | 7 antigen panel*  72.9%  * ANGPTL4,  DKK, GAL, MUC1,  GFRA, GRN LRRC15 | 76% | ANGPTL4 – 1.57 (1.24 – 1.99)  DKK1 - 1.77 (1.4 – 2.24)  EPHA2 - 1.64 (1.21- 2.24)  GAL1 – 1.75 (1.37 – 2.23)  HER-2 – 1.65 (1.28 – 2.13)  IGFBP2 – 1.39 (1.10 – 1.75) P<0.001  LAMC2 – 1.47 (1.16 – 1.88)  MUC1 – 1.83 (1.41 – 2.37)  SPON2 – 1.65 (1.31 – 2.07)  CST2 – 1.39 (1.12 – 1.73)  SPINT2 – 1.4 (1.13 – 1.74)  SSR2 – 1.53 (1.23 – 1.92) | 7 antigen panel*  8.56 |  |
|  | Fernandez Madrid et al., 2004 [63] | Clinical data/ biopsy proven diagnosis – patient group  Unclear – healthy group | Microarray technique | A positive cut-off value was determined for each serum as CY3/CY5 ratio of 3 SDs above the mean ratio of the 938 signals. | Training set – 76%, Independent set – 78%,  combined set – 77%  Annexin XI-A  19% | Training set – 92%.,  Independent set – 84%, combined set – 88%  Annexin XI-A  98% |  | For one or more of the 12 antigens  Training set – 37.1  Independent set – 18.3  Combined set – 24.64  Annexin XI-A  11.64 |  |
|  | Fernandez Grijalva et al., 2014, Mexico [50] | Mammography – patient group  Comprehensive medical check –up – healthy group | Immune-proteomics | Unclear | 91.7% | 91.7% |  | 122.2 |  |
|  | Frenkel K et al., 1998  New York [51] | Unclear | ELISA | Unclear |  |  | OR based on ab titre  <10 – 18.9 – 3.59  19 – 36.4 – 1.96  >36.5 – 11.68 |  |  |
|  | Gao et al., 2005, China [30] | Unclear | ELISA | P53 index = OD450 nm absorbance of a sample/OD450 nm absorbance of the serum control. Serum samples with a P53 index >1.2 were considered positive. | 21.5% | 95% |  | 5.47 |  |
|  | Hamrita et al., 2008, Tunisia [44] | Histological diagnosis – patient group  Routine health check-up – healthy group | 2d immunoblot, IHC | Unclear | Frequency of SIGNIFICANT antibodies given and were translated into sensitivity and specificity values by the reviewer  HSP60 – 48%, PHB2- 45%, beta tubulin chain – 43%, PRDX2 – 38%, haptoglobulin – 40% | HSP60 – 95%, PHB2- 93%, beta tubulin chain – 95%, PRDX2 – 95%, haptoglobulin – 95% |  | HSP60 -18.1  PHB2 – 10.63  beta tubulin chain – 14.8  PRDX2 – 14.8  Haptoglobulin – 13.3 |  |
|  | Huang et al.,2015, China [45] | Mammographic and radiographic examination – patient group  Clinical interview and radiographic examination – healthy group | ELISA | Unclear | 23.2% | 90% |  | 2.13 |  |
|  | Kyo Yi et al., 2009, Korea [27] | Unclear – patient group  Comprehensive medical check-up – healthy group | 2D immunoblot  analysis | Unclear | 79.1% | 90.4% |  | 35.4 |  |
|  | Kulic et al., 2009, Croatia [31] | Unclear | ELISA | The cut-off was calculated by multiplying the absorbance (OD 450nm) of the undiluted calibrator with the cut off specific factor printed on the calibrator bottle. The cut off value was defined as 15 U/ml | Frequency of antibodies given and were translated into sensitivity and specificity values by the reviewer  35% | 95% |  | 9.97 |  |
|  | Lacombe et al., 2013, France [64] | Histopathologic diagnosis – patient group  Negative mammogram, negative physical breast exam – healthy group | ELISA | Mean optical density of normal samples +2SD of mean. | Validation set  GAL3 -32%  PAK2 – 25%  PHB2 – 24%  RACK1 – 31%  RUVBL1 – 24% | Validation set  GAL3 -94%  PAK2 – 96%  PHB2 – 97%  RACK1 – 94%  RUVBL1 – 94% |  | Validation set  GAL3 -8.4  PAK2 – 7.2  PHB2 – 10.44  RACK1 – 7.11  RUVBL1 – 5.06 |  |
|  | Lacombe et al., 2014, France [32] | Histopathologic diagnosis – patient group  Negative mammogram – healthy group | ELISA | Absorbance greater than 2SDs above the mean value of the controls. | The specificities of autoantibodies were tested by keeping a maximum sensitivity of 90%, 95% and 99%  (Supplementary Annexure 7) |  |  |  |  |
|  | Naour et al., 2001, France [52] | Unclear | Western blot,  IHC | Unclear | 37%  (Frequency of autoantibodies given from which sensitivity value was computed by the reviewer) | Unclear |  |  |  |
|  | Lenner et al., 1999, Sweden [33] | Unclear | ELISA, Western blot | The mean of the case mean and control mean was used as lower cut-off, and the mean of the controls plus two standard deviations as upper cut-off |  |  | ELISA:  9.03; 95% CI (2.40 – 50.43) |  |  |
|  | Liu, T et al., 2014, China [23] | Radiological and histological confirmation – patient group  Radiological and clinical interview – healthy group | ELISA | Unclear | CD25 – 98%  FOXP3 – 99.3% | CD25 – 2%  FOXP3 – 9% |  | CD25 – 0.9  FOXP3 -15.71 |  |
|  | Liu W et al., 2015, Texas [47] | Unclear – patient group  Annual health examination – healthy group | ELISA | The cut-off value for determining a positive reaction was designated as the mean OD value of the 44 normal human sera plus 3 standard deviations (mean + 3SD). | 14.3% | 98% |  | 7 |  |
|  | Liu W et al., 2015, Texas [46] | Unclear – patient group  Annual health examination – healthy group | ELISA, Western blot | The cut off value for determining a positive reaction was designated as the mean OD value of the 38 normal human sera plus 2 standard deviations (mean+2 SD). | Cyclin B1- 18.4 %,  Imp1- 18.4 %, Koc - 16.3 %, survivin - 16.3 %,  p16- 14.3%,  c-Myc - 12.2 %  Panel – 67.3% | Cyclin B1- 97.6%, Imp1- 100 %, Koc – 97.6 %, survivin – 97.6 %, p16  - 100%), c-Myc - 100%  Panel – 92.1% |  | Cyclin B1- 8.3  Imp1- 18.06  Koc – 7.21 survivin – 7.21  p16- 13.5  c-Myc – 11.5  Panel – 24.1 | Positive predictive value (PPV) – 91.7%  Negative predictive value (NPV) - 68.6% |
|  | Liu X et al., 2014, USA [65] | Mammography and pathological diagnosis | ELISA, IHC, IFC | Mean OD of 90 normal human sera plus 3SDs | 19% | 98% |  | 10.1 |  |
|  | Liu Y et al., 2017, China [34] | radiographic examination and histology – patient group  clinical interviews and imaging examinations – healthy group | ELISA | Unclear | P16 – 27.5%, c- Myc – 11.8%, TP53 – 24.5%, ANXA-1 – 17.6%  Panel – 33.3% | Sensitivity calculated against a fixed specificity of 90% |  |  | AUC values  P16 – 0.575, c- Myc – 0.574, TP53 – 0.705, ANXA-1 – 0.733 |
|  | Lopez et al., 2012, Mexico [66] | Biopsy – patient group  Comprehensive medical check-up and mammogram – healthy group | 2D electrophoresis, Western blot (immuneproteomic approach) | Absorbance greater than 2SDs above the mean value of the controls. | 96% | 90% |  | 240 |  |
|  | Lu H et al., 2012, USA [35] | Unclear – patient group  5-year follow-up period – healthy group | ELISA, protein microarrays | Absorbance greater than 2SDs above the mean value of the controls. | Distant from diagnosis:  P53 – 22%, HER2 – 17%, CEA – 26%, cyc B1 – 12%  Pre-diagnostic:  P53 – 6%, HER2- 15% | Distant from diagnosis:  P53 – 97%, HER2 – 94%, CEA – 87%, cyc B1 – 97%  Pre-diagnostic:  P53 – 100%, HER2- 100% |  | Distant from diagnosis  P53 – 9.16  HER2 – 3.21  CEA – 2.23 cyc B1 – 4.41  Pre-diagnostic:  P53 – 7.22 HER2 – 17.56 | In newly diagnosed patients - 0.73 (95% CI: 0.56 to 0.87, p=0.018). |
|  | Mohammed et al., 2015  Saudi Arabia [24] | Unclear | ELISA | Unclear | ANA – 100%  ASDS – 2.9%  (computed by the reviewer) | Unclear |  |  |  |
|  | Nunna et al., 2014, India [17] | Unclear | ELISA, SDS-PAGE | Autoantibody titers were arbitrary expressed as a mean absorbance values = 0.257 was considered positive. | Not mentioned for breast cancer separately | 76.3% |  |  |  |
|  | Oleg et al., 2018, Estonia [47] | Histological diagnosis of breast cancer | ELISA | Unclear | 75% | 82% |  |  | AUC – 0.64 |
|  | Olga et al., 2017, Russia [67] | Histological findings to confirm cancer | ELISA, SEREX, qPCR | Mean of healthy group + 3SD | RAD50 – 9.29%, PARD3 – 32.14%, SPP1- 41.07%, SAP30BP – 33.04%, NY-BR-62- 13.39%, NY-CO-58 – 12.50%  Panel – 70% | RAD50 – 100%, PARD3 – 88.57%, SPP1- 85.71%, SAP30BP – 91.43%, NY-BR-62- 97.14%, NY-CO-58 – 97.14%  Panel – 91% |  |  | AUC – 0.945 |
|  | Pagaza-Straffon et al.  2020, Mexico [36] | Clinical diagnosis (?) | ELISA | Mean + 2SD of controls | CEA – 1.9%, c-Myc – 3.8%, p53 – 4.8%, Ki-67 – 2.88%, Nm23 – 2.88%, PRDX6 – 3.8%, eIF5A – 1.9%, GLIO-1- 0.96%, Hsp70 – 2.88%  p53/PRDX6/CEA – 19%,  p53/PRDX6/c-Myc/Hsp70 – 28%  p53/PRDX6/c-Myc/Hsp70/Nm23 – 24% | CEA – 96%, c-Myc – 98%, p53 – 100%, Ki-67 – 98%, Nm23 – 98%, PRDX6 – 98%, eIF5A – 96%, GLIO-1- 96%, Hsp70 – 96%  100% for all 3 panels |  |  |  |
|  | Qiu et al., 2019  China [37] | Clinical diagnosis (?) | ELISA, western blotting | maximum Youden index when the specificity was greater than 95% | Panel:  p53, cyclinB1, p16, p62, 14-3-3ξ  78.2% | Panel:  p53, cyclinB1, p16, p62, 14-3-3ξ  89% |  |  |  |
|  | Regele et al., 1999, Germany [19] | Histological diagnosis | ELISA | Unclear | 11.6%  (Frequency of autoantibodies given from which sensitivity value was computed by the reviewer) |  |  |  |  |
|  | Tang et al., 2010, China [41] | Unclear | ELISA | Mean OD value plus three standard deviations (SD) for anti-MUC1 IgG and the mean OD value plus two SD for anti-MUC1 IgM. | MUC1 IgG – 50%, MUC1 IgM – 25% | MUC1 IgG – 76.8%, MUC1 IgM – 74.4% |  | MUC1 IgG – 3.3  MUC1 IgM – 0.97 |  |
|  | Tomkiel et al., 2002  Detroit, USA [13] | Unclear | ELISA, IHC | To determine positive reactivity, we chose as our cut-off value a final ELISA value of 0.425. This value represents 3 SDs above the average for the 65 non-cancer control sera and allowed clear differentiation between positive and negative sera | 10.9%  (Frequency/ prevalence of autoantibodies given from which sensitivity value was computed by the reviewer) | 100% |  |  |  |
|  | Wandall et al., 2010, UK [18] | Unclear | Microarray, ELISA | Values were considered positive if above three times the SD of the average value obtained with sera from healthy individuals | 19.23%  (Frequency/ prevalence of autoantibodies given from which sensitivity value was computed by the reviewer) | Specificity: ‘low’ |  |  |  |
|  | Wang J et al., 2015, Poland [25] | Pathology information | ELISA, NAPPA arrays | Unclear | 13 antigen panel  33% | 13 antigen panel  98% |  | 22.52 | ROC analysis  showed the 13 AAb classifier had an AUC of 0.68 (95% CI, 0.67–  0.70 |
|  | Wu et al., 2009  China [14] | Unclear – patient group  Routine physical examination – healthy group | ELISA | Positivity was defined as (sample OD450 value)/ (negative control OD450 value) >2 | 16%  (sensitivity and specificity for breast cancer) | 98.98% |  | 18.41 | PPV – 93.5%  NPV – 53.7% |
|  | Yagihashi et al., 2005, Japan [68] | Unclear | ELISA | Cut- off:  Mean absorbance +2SD for healthy control samples  Anti – survivin - 0.441 at absorbance 492 nm,  Anti – livin – 0.413 at absorbance 492 nm | Survivin – 23.9%,  livin – 32.6%  (Frequency/ prevalence of autoantibodies given from which sensitivity value was computed by the reviewer) | Unclear |  |  |  |
|  | Yahalom et al., 2013, USA [26] | Pathological evaluation | ELISA | A cancer patient is defined when AAb B > AAb A, and a healthy individual is defined when AAb B < AAb A, where  AAb B - cancer-related AAbs  AAb A - normal amounts of AAbs | Whole population of 507 samples – 49.5%  Training set of 204 samples – 61.8%  Prediction set of 48 samples – 45.4% | Whole population of 507 samples – 95.2% (CI = 92.8 – 96.8)  Training set of 204 samples – 94.7% (CI = 88.0 – 98.3)  Prediction set of 48 samples – 100% (CI = 78.2 – 100.0) |  | Whole population – 19.3  Training set – 28.8  Prediction set – 25.97 | Whole population – 0.8  Prediction set – 0.8  Training set – 0.84 |
|  | Ye et al., 2012  China [38] | Clinical information | ELISA, Western blot | Mean absorbance of the 82 normal human sera (NHS) plus 2 standard deviations (mean + 2SD). | c‑myc -22.0%, survivin - 22.0%  Cyclin B1- 17.1%,  cyclin D1- 17.1%,  p62- 12.2%, P53 -12.2%, p16- 12.2%, Imp1- 12.2%, CDK2 - 9.8% Koc 7.3%.  Panel:  c‑myc, survivin, Cyclin B1, cyclin D1, p62, P53, p12 and CDK2 – 61.0% | c‑myc -100.0%, survivin – 98.7%  Cyclin B1- 98.7%, cyclin D1- 97.6%,  p62- 98.7%, P53 -97.6%, p16- 97.6%, Imp1-97.6%,  CDK2 -98.7%  Koc 98.7%.  Panel:  c‑myc, survivin, Cyclin B1, cyclin D1, p62, P53, p12 and CDK2 – 89.0% |  | c‑myc – 48.23  survivin – 22.7  Cyclin B1 – 16.6  cyclin D1 – 8.2  p62 – 11.25  P53 – 5.55  p12 – not mentioned  Panel – 12.67 | PPV: 73.5% (for 8 antigens)  NPV: 82.0% |
|  | Zhong et al., 2008, China [54] | Histologic confirmation – patient group  Unclear for healthy group | ELISA | Unclear | From logistic regression analysis  KLF 17 – 28%, COL6A1- 33%, GRWD1- 34%, ASB-9 -41%, SERAC1- 57%, RELT – 53% | From logistic regression analysis  KLF 17 – 100%, COL6A1- 100%, GRWD1- 100%, ASB-9 - 100%, SERAC1- 100%, RELT – 100% |  |  |  |
|  | Zhu et al., 2015, USA [69] | Histological confirmation – patient group  Annual health examination – healthy group | ELISA | A positive test for antibodies was taken as an absorbance reading above the mean+3SD of the normal | PARP1- 15.3%, BRCA1- 19.1%, BRCA2 - 36.6%  Panel:  PARP1 and BRCA1 - 5.3%;  PARP1 and BRCA2- 7.6%;  BRCA1 and BRCA2 - 4.6%,  PARP1, BRCA1 and BRCA2 - 4.6%  (Frequency/ prevalence of autoantibodies given from which sensitivity value was computed by the reviewer) | PARP1- 99.25%, BRCA1- 99.25%, BRCA2 -99.25%,  Panel:  PARP1 and BRCA1 – 100%;  PARP1 and BRCA2- 100%;  BRCA1 and BRCA2 - 100%;  PARP1, BRCA1 and BRCA2 - 100% |  | PARP1- 24.14 BRCA1- 31.6 BRCA2 – 77.5 |  |
|  | Zuo et al., 2014, China [42] | Unclear | Biopanning, ELISA | Unclear | LGALS3 – 63%, Phb2 – 43%, MUC1- 43%, GK2 – 46%, CA 15-3 – 58%  Panel – 87% | Panel – 76%  No individual specificities mentioned |  | 1.23 | ROC:  LGALS3- 0.687, PHB2 - 0.583, MUC1- 0.563, CA15-3 - 0.634, and  GK2 -0.608  Panel – 0.872 |

*Additional references (not cited in main text)*

62. Anderson KS, Sibani S, Wallstrom G, Qiu J, Mendoza EA, Raphael J, et al. Protein microarray signature of autoantibody biomarkers for the early

detection of breast cancer. J Proteome Res. 2011. https:// doi. org/ 10. 1021/pr100 686b.

63. Fernandez-Madrid F, Tang N, Alansari H, Granda JL, Tait L, Amirikia KC, et al. Autoantibodies to Annexin XI-A and other autoantigens in the

diagnosis of breast cancer. Cancer Res. 2004. https:// doi. org/ 10. 1158/0008- 5472. CAN- 03- 0932.

64. Lacombe J, Mange A, Jarlier M, Bascoul-Mollevi C, Rouanet P, Lamy P-J, et al. Identification and validation of new autoantibodies for the diagnosis

of DCIS and node negative early-stage breast cancers. Int J Cancer. 2013. https:// doi. org/ 10. 1002/ ijc. 27766.

65. Liu X, Chai Y, Li J, Ren P, Liu M, Dai L, et al. Autoantibody response to a novel tumor-associated antigen p90/CIP2A in breast cancer immunodiagnosis.

Tumour Biol. 2014. https:// doi. org/ 10. 1007/ s13277- 013- 1350-6.

66. Lopez-Arias E, Aguilar-Lemarroy A, Felipe Jave-Suarez L, Morgan-Villela G, Mariscal-Ramirez I, Martinez-Velazquez M, et al. Alpha 1-antitrypsin: a

novel tumor-associated antigen identified in patients with early-stage breast cancer. Electrophoresis. 2012. https:// doi. org/ 10. 1002/ elps. 201100491.

67. Kostianets O, Shyyan M, Antoniuk SV, Filonenko V, Kiyamova R. Panel of SEREX-defined antigens for breast cancer autoantibodies profile detection.

Biomarkers. 2017. https:// doi. org/ 10. 1080/ 13547 50X. 2016. 12529 52.

68. Yagihashi A, Ohmura T, Asanuma K, Kobayashi D, Tsuji N, Torigoe T, et al. Detection of autoantibodies to survivin and livin in sera from patients

with breast cancer. Clin Chim Acta. 2005. https:// doi. org/ 10. 1016/j. cccn.2005. 06. 009.

69. Zhu Q, Han S-X, Zhou C-Y, Cai M-J, Dai L-P, Zhang J-Y. Autoimmuneresponse to PARP and BRCA1/BRCA2 in cancer. Oncotarget. 2015. https://

doi. org/ 10. 18632/ oncot arget. 3428.
