# Supplementary material for: Can serum autoantibodies be a potential early detection biomarker for breast cancer in women? A diagnostic test accuracy review and meta-analysis
Source: Syst Rev. 2022 Oct 9;11:215. doi: 10.1186/s13643-022-02088-y (PMC9549667; doi:10.1186/s13643-022-02088-y)
Supplement: Supplementary file 6 — Additional file 6. Autoantibodies. [file 13643_2022_2088_MOESM6_ESM.docx]

Autoantibodies

1. ATP6AP1
2. p53
3. MUC1
4. Recombinant mammoglobin
5. Native mammoglobin -lipophillin B complex and
6. Lipophillin B
7. c-myc
8. HER2
9. NY-ESO-1
10. BRCA1
11. BRCA2
12. p16
13. Astrocyte elevated gene – 1 (AEG -1)
14. ECPKA (extracellular protein kinase A)
15. HSP60
16. hnRNPF
17. FTH1
18. PPIA
19. FKBP52
20. ANGPTL4
21. DKK1
22. EPHA2
23. GAL1
24. IGFBP2
25. LAMC2
26. SPON2
27. CST2
28. SPINT2
29. SSR2
30. Annexin XI – A
31. Alpha 2HS glycoprotein
32. Anti- HMdU
33. LGALS3BP (lectin galactoside –binding soluble 3 binding protein)
34. alpha B crystalline
35. hnRNPK
36. hnRNPH3
37. beta tubulin
38. prohibitin
39. Mn-SOD
40. PD1
41. ANXA1
42. GAL 3
43. PAK 2
44. RACK1
45. RUVBL1
46. PRDX2
47. CCNB1
48. RS/ DJ – 1
49. CD25
50. FOXP3
51. IMP2/p62
52. Imp1
53. p16
54. Koc
55. Survivin
56. cyclin B1
57. p90/CIP2A
58. Alpha 1 antitrypsin
59. topo 2 alpha
60. CEA
61. CatD
62. Hyaluronic acid binding proteins
63. MAGE-1
64. MAGE-3SSX2
65. Melan-A
66. Tyrosinase protein
67. carbonic anhydrase
68. p63
69. p73
70. RPA32 (replication protein A)
71. livin
72. cyclin D1
73. p62
74. p12
75. CDK2,
76. KLF 17
77. COL6A1
78. GRWD1
79. ASB-9
80. SERAC1
81. RELT
82. PARP1
83. LGALS3
84. Phb2
85. GK2
86. TriosePhosphate Isomerase 1 (TPI1)
87. Thomsen-Friedenreich Antigen-Specific Antibody
88. RAD50
89. PARD3
90. SPP1
91. SAP30BP
92. NY-BR-62
93. NY-CO-58
94. Ki-67
95. Nm23
96. PRDX6
97. eIF5A
98. GLIO-1
99. Hsp70
100. 14-3-3ξ
