# Supplementary material for: Can serum autoantibodies be a potential early detection biomarker for breast cancer in women? A diagnostic test accuracy review and meta-analysis
Source: Syst Rev. 2022 Oct 9;11:215. doi: 10.1186/s13643-022-02088-y (PMC9549667; doi:10.1186/s13643-022-02088-y)
Supplement: Supplementary file 7 — Additional file 7. Threshold and methods of analyses. [file 13643_2022_2088_MOESM7_ESM.docx]

Threshold and methods of analyses

| **> Mean plus 2SD of the normal cohort** | | **> Mean plus 3 SD of the normal cohort** | | **> Mean plus 4 SD of the normal cohort** | |
| --- | --- | --- | --- | --- | --- |
| *Author and autoantibodies* | *Method of analysis* | *Author and autoantibodies* | *Method of analysis* | *Author and autoantibodies* | *Method of analysis* |
| **Anderson K S**  ATP6ATP | NAPPA (Novel high density custom protein microarray) and ELISA | **Carter, 2003**  Lipophillin B |  | **Blixt Ola, 2011**  MUC 1 | microaaray |
| **Chapman 2007**  p53, c-myc, HER2, NY-ESO-1, BRCA1, BRCA2 and MUC1 | ELISA | **Liu W, 2015**  IMP2/P62 | ELISA |  | |
| **Desmetz C, 2008**  HSP60 | ELISA | **Liu X, 2014**  p90/CIP2A | ELISA |  | |
| **Evans, 2014**  ANGPTL4, DKK1, EPHA2, GAL1, HER-2, IGFBP2, LAMC2, MUC1, SPON2, CST2, SPINT2 and SSR2 | conformation carrying antigen ELISA | **Stockhert, 1998**  NY-ESO1, MAGE-1, MAGE-3, SSX2, Melan-A, Tyrosinase protein |  |  | |
| **Grassadonia, 2013**  LGALS3BP |  | **Tomkiel, 2009**  RPA -32 antibody | ELISA |  | |
| **Lacombe J, 2013**  GAL3, PAK2, PHB2, RACK1 and RUVBL1 | ELISA | **Wandall, 2010**  MUC-1 | Microarray |  | |
| **Lacombe J, 2014**  HSP60, FKBP52, PRDX2, PP1A, MUC1, GAL3, PAK2, P53, CCNB1, PHB2, RACK1, RUVBL1 and HER2 | ELISA | **Zhu, 2015**  PARP, BRCA1, BRCA2 | ELISA |  | |
| **Liu W, 2015**  Imp1, p16, Koc,  survivin, cyclin B1, and c-myc | ELISA |  | |  | |
| **Lopez Arias , 2012**  Alpha 1 antitrypsin | 2DE, Western Blot, MALDI – MS |  | |  | |
| **Lu. H, 2012**  HER-2/neu, p53, topo 2 alpha, MUC1, CEA, catD and cyclin B1 | ELISA |  | |  | |
| **Ye.H, 2013**  Imp1, p62, Koc, p53, c-myc  , survivin, p16, cyclin B1, cyclin D1 and CDK2 | ELISA |  | |  | |
