# Supplementary material for: Can serum autoantibodies be a potential early detection biomarker for breast cancer in women? A diagnostic test accuracy review and meta-analysis
Source: Syst Rev. 2022 Oct 9;11:215. doi: 10.1186/s13643-022-02088-y (PMC9549667; doi:10.1186/s13643-022-02088-y)
Supplement: Supplementary file 8 — Additional file 8. Study and 2 × 2 tables. [file 13643_2022_2088_MOESM8_ESM.docx]

|  | |
| --- | --- |
| Sl. No | Study and 2 X 2 tables |
|  | Anderson K S et al., 2011   \| Panel \| patients \| healthy \| Total \| \| --- \| --- \| --- \| --- \| \| + \| 45 \| 3 \| 48 \| \| - \| 57 \| 74 \| 131 \| \| Total \| 102 \| 77 \| 179 \|  \| ATP6AP1 \| patients \| healthy \| Total \| \| --- \| --- \| --- \| --- \| \| + \| 19 \| 3 \| 22 \| \| - \| 129 \| 61 \| 190 \| \| Total \| 148 \| 64 \| 212 \| |
|  | Angelopoulou et al., 1994   \| P53 \| patients \| healthy \| Total \| \| --- \| --- \| --- \| --- \| \| + \| 10 \| 1 \| 11 \| \| - \| 280 \| 229 \| 509 \| \| Total \| 290 \| 230 \| 520 \| |
|  | Balogh et al., 2005   \| P53 \| patients \| healthy \| Total \| \| --- \| --- \| --- \| --- \| \| + \| 9 \| 0 \| 9 \| \| - \| 46 \| 8 \| 54 \| \| Total \| 55 \| 8 \| 63 \| |
|  | Chapman et al. 2007   \| P53 \| PBC  patients \| healthy \| Total \| \| --- \| --- \| --- \| --- \| \| + \| 23 \| 4 \| 27 \| \| - \| 74 \| 90 \| 164 \| \| Total \| 97 \| 94 \| 191 \|  \| NY-ESO-1 \| PBC  patients \| healthy \| Total \| \| --- \| --- \| --- \| --- \| \| + \| 25 \| 6 \| 31 \| \| - \| 72 \| 88 \| 160 \| \| Total \| 97 \| 94 \| 191 \|  \| c-Myc \| PBC  patients \| healthy \| Total \| \| --- \| --- \| --- \| --- \| \| + \| 13 \| 3 \| 16 \| \| - \| 84 \| 91 \| 175 \| \| Total \| 97 \| 94 \| 191 \|  \| BRCA2 \| PBC  patients \| healthy \| Total \| \| --- \| --- \| --- \| --- \| \| + \| 33 \| 8 \| 41 \| \| - \| 64 \| 86 \| 150 \| \| Total \| 97 \| 94 \| 191 \|  \| BRCA1 \| PBC  patients \| healthy \| Total \| \| --- \| --- \| --- \| --- \| \| + \| 8 \| 8 \| 16 \| \| - \| 89 \| 86 \| 175 \| \| Total \| 97 \| 94 \| 191 \|  \| HER2 \| PBC  patients \| healthy \| Total \| \| --- \| --- \| --- \| --- \| \| + \| 17 \| 6 \| 23 \| \| - \| 80 \| 88 \| 168 \| \| Total \| 97 \| 94 \| 191 \|  \| MUC1 \| PBC  patients \| healthy \| Total \| \| --- \| --- \| --- \| --- \| \| + \| 19 \| 2 \| 21 \| \| - \| 78 \| 92 \| 170 \| \| Total \| 97 \| 94 \| 191 \|  \| Panel \| PBC  patients \| healthy \| Total \| \| --- \| --- \| --- \| --- \| \| + \| 62 \| 14 \| 76 \| \| - \| 35 \| 80 \| 115 \| \| Total \| 97 \| 94 \| 191 \|      \| P53 \| DCIS  patients \| healthy \| Total \| \| --- \| --- \| --- \| --- \| \| + \| 6 \| 4 \| 10 \| \| - \| 34 \| 90 \| 124 \| \| Total \| 40 \| 94 \| 134 \|  \| c-Myc \| DCIS  patients \| healthy \| Total \| \| --- \| --- \| --- \| --- \| \| + \| 3 \| 3 \| 6 \| \| - \| 37 \| 91 \| 128 \| \| Total \| 40 \| 94 \| 134 \|  \| NY-ESO-1 \| DCIS  patients \| healthy \| Total \| \| --- \| --- \| --- \| --- \| \| + \| 3 \| 6 \| 9 \| \| - \| 37 \| 88 \| 125 \| \| Total \| 40 \| 94 \| 134 \|        \| BRCA2 \| DCIS  patients \| healthy \| Total \| \| --- \| --- \| --- \| --- \| \| + \| 9 \| 8 \| 17 \| \| - \| 31 \| 86 \| 117 \| \| Total \| 40 \| 94 \| 134 \|  \| BRCA1 \| DCIS  patients \| healthy \| Total \| \| --- \| --- \| --- \| --- \| \| + \| 1 \| 8 \| 9 \| \| - \| 39 \| 86 \| 125 \| \| Total \| 40 \| 94 \| 134 \|  \| HER2 \| DCIS  patients \| healthy \| Total \| \| --- \| --- \| --- \| --- \| \| + \| 5 \| 6 \| 11 \| \| - \| 35 \| 88 \| 123 \| \| Total \| 40 \| 94 \| 134 \|      \| MUC1 \| DCIS  patients \| healthy \| Total \| \| --- \| --- \| --- \| --- \| \| + \| 9 \| 2 \| 11 \| \| - \| 31 \| 92 \| 123 \| \| Total \| 40 \| 94 \| 134 \|  \| Panel \| DCIS  patients \| healthy \| Total \| \| --- \| --- \| --- \| --- \| \| + \| 18 \| 14 \| 32 \| \| - \| 22 \| 80 \| 102 \| \| Total \| 40 \| 94 \| 134 \| |
|  | Chen C et al, 2014.   \| P16 \| patients \| healthy \| Total \| \| --- \| --- \| --- \| --- \| \| + \| 66 \| 16 \| 82 \| \| - \| 86 \| 144 \| 230 \| \| Total \| 152 \| 160 \| 312 \| |
|  | Chen X et al, 2012.   \| AEG1 \| patients \| healthy \| Total \| \| --- \| --- \| --- \| --- \| \| + \| 44 \| 0 \| 44 \| \| - \| 54 \| 115 \| 169 \| \| Total \| 98 \| 115 \| 213 \| |
|  | Nestorva et al 2006  For all types of cancers   \| ECPKA \| patients \| healthy \| Total \| \| --- \| --- \| --- \| --- \| \| + \| 309 \| 21 \| 330 \| \| - \| 34 \| 142 \| 176 \| \| Total \| 343 \| 163 \| 506 \| |
|  | Desmetz et al. 2008   \| HSP60 \| Early stage  patients \| healthy \| Total \| \| --- \| --- \| --- \| --- \| \| + \| 18 \| 4 \| 22 \| \| - \| 40 \| 89 \| 129 \| \| Total \| 58 \| 93 \| 151 \|  \| HSP60 \| DCIS  patients \| healthy \| Total \| \| --- \| --- \| --- \| --- \| \| + \| 16 \| 4 \| 20 \| \| - \| 33 \| 89 \| 122 \| \| Total \| 49 \| 93 \| 1 \| |
|  | Dong et al, 2013   \| hnRNPF \| patients \| healthy \| Total \| \| --- \| --- \| --- \| --- \| \| + \| 130 \| 60 \| 190 \| \| - \| 25 \| 95 \| 120 \| \| Total \| 155 \| 155 \| 310 \|  \| FTH1 + hnRNPF \| patients \| healthy \| Total \| \| --- \| --- \| --- \| --- \| \| + \| 141 \| 43 \| 184 \| \| - \| 14 \| 112 \| 123 \| \| Total \| 155 \| 155 \| 310 \|  \| FTH1 \| patients \| healthy \| Total \| \| --- \| --- \| --- \| --- \| \| + \| 126 \| 68 \| 194 \| \| - \| 29 \| 87 \| 116 \| \| Total \| 155 \| 155 \| 310 \|  \| FTH1 + hnRNPF + CA15-3 \| patients \| healthy \| Total \| \| --- \| --- \| --- \| --- \| \| + \| 138 \| 9 \| 147 \| \| - \| 17 \| 146 \| 163 \| \| Total \| 155 \| 155 \| 310 \| |
|  | Desmetz et al. 2009   \| PPIA \| patients \| healthy \| Total \| \| --- \| --- \| --- \| --- \| \| + \| 71 \| 12 \| 83 \| \| - \| 71 \| 81 \| 152 \| \| Total \| 142 \| 93 \| 235 \|  \| FKBP52 \| patients \| healthy \| Total \| \| --- \| --- \| --- \| --- \| \| + \| 71 \| 12 \| 83 \| \| - \| 71 \| 81 \| 152 \| \| Total \| 142 \| 93 \| 235 \|  \| MUC1 \| patients \| healthy \| Total \| \| --- \| --- \| --- \| --- \| \| + \| 53 \| 11 \| 64 \| \| - \| 89 \| 82 \| 171 \| \| Total \| 142 \| 93 \| 235 \|  \| PRDX2 \| patients \| healthy \| Total \| \| --- \| --- \| --- \| --- \| \| + \| 64 \| 13 \| 77 \| \| - \| 78 \| 80 \| 158 \| \| Total \| 142 \| 93 \| 235 \|  \| HSP60 \| patients \| healthy \| Total \| \| --- \| --- \| --- \| --- \| \| + \| 50 \| 12 \| 62 \| \| - \| 92 \| 81 \| 173 \| \| Total \| 142 \| 93 \| 235 \| |
|  | Evans et al, 2014   \| 7 ag panel \| patients \| healthy \| Total \| \| --- \| --- \| --- \| --- \| \| + \| 146 \| 48 \| 194 \| \| - \| 54 \| 152 \| 206 \| \| Total \| 200 \| 200 \| 400 \| |
|  | \| Annexin XI-A \| patients \| healthy \| Total \| \| --- \| --- \| --- \| --- \| \| + \| 17 \| 1 \| 18 \| \| - \| 73 \| 50 \| 123 \| \| Total \| 90 \| 51 \| 141 \|   Fernandes Madrid 2004   \| 1 or more of 12 antigens \| patients \| healthy \| Total \| \| --- \| --- \| --- \| --- \| \| + \| 69 \| 6 \| 75 \| \| - \| 21 \| 45 \| 66 \| \| Total \| 90 \| 51 \| 141 \|  \| 1 or more of 12 antigens \| patients \| healthy \| Total \| \| --- \| --- \| --- \| --- \| \| + \| 34 \| 2 \| 36 \| \| - \| 11 \| 24 \| 35 \| \| Total \| 45 \| 26 \| 71 \|  \| 1 or more of 12 antigens \| patients \| healthy \| Total \| \| --- \| --- \| --- \| --- \| \| + \| 35 \| 4 \| 39 \| \| - \| 10 \| 21 \| 31 \| \| Total \| 45 \| 25 \| 70 \| |
|  | Fernandes Grijalva, 2014   \| Alpha 2HS GP \| patients \| healthy \| Total \| \| --- \| --- \| --- \| --- \| \| + \| 33 \| 3 \| 36 \| \| - \| 3 \| 33 \| 36 \| \| Total \| 36 \| 36 \| 72 \| |
|  | Gao et al, 2005   \| P53 \| patients \| healthy \| Total \| \| --- \| --- \| --- \| --- \| \| + \| 32 \| 12 \| 44 \| \| - \| 112 \| 230 \| 342 \| \| Total \| 144 \| 242 \| 386 \| |
|  | Hamrita et al. 2008.   \| PRDX2 \| patients \| healthy \| Total \| \| --- \| --- \| --- \| --- \| \| + \| 15 \| 2 \| 17 \| \| - \| 25 \| 40 \| 65 \| \| Total \| 40 \| 42 \| 82 \|  \| HSP60 \| patients \| healthy \| Total \| \| --- \| --- \| --- \| --- \| \| + \| 19 \| 2 \| 21 \| \| - \| 21 \| 40 \| 61 \| \| Total \| 40 \| 42 \| 82 \|  \| PHB2 \| patients \| healthy \| Total \| \| --- \| --- \| --- \| --- \| \| + \| 18 \| 3 \| 21 \| \| - \| 22 \| 39 \| 61 \| \| Total \| 40 \| 42 \| 82 \|  \| tubulin chain \| patients \| healthy \| Total \| \| --- \| --- \| --- \| --- \| \| + \| 17 \| 2 \| 19 \| \| - \| 23 \| 40 \| 63 \| \| Total \| 40 \| 42 \| 82 \|  \| Haptoglobulin \| patients \| healthy \| Total \| \| --- \| --- \| --- \| --- \| \| + \| 16 \| 2 \| 18 \| \| - \| 24 \| 40 \| 64 \| \| Total \| 40 \| 42 \| 82 \| |
|  | Huang et al,2015   \| ANXA 1 \| patients \| healthy \| Total \| \| --- \| --- \| --- \| --- \| \| + \| 35 \| 16 \| 51 \| \| - \| 117 \| 144 \| 261 \| \| Total \| 152 \| 160 \| 312 \| |
|  | Kyo Yi et al,2009   \| 2HS GP \| patients \| healthy \| Total \| \| --- \| --- \| --- \| --- \| \| + \| 64 \| 7 \| 71 \| \| - \| 17 \| 66 \| 83 \| \| Total \| 81 \| 73 \| 154 \| |
|  | Kulic et al,2009   \| P53 \| patients \| healthy \| Total \| \| --- \| --- \| --- \| --- \| \| + \| 21 \| 1 \| 22 \| \| - \| 40 \| 19 \| 59 \| \| Total \| 61 \| 20 \| 81 \| |
|  | Lacombe et al, 2013  Validation set   \| GAL3 \| patients \| healthy \| Total \| \| --- \| --- \| --- \| --- \| \| + \| 36 \| 4 \| 40 \| \| - \| 68 \| 64 \| 132 \| \| Total \| 104 \| 68 \| 172 \|  \| PAK2 \| patients \| healthy \| Total \| \| --- \| --- \| --- \| --- \| \| + \| 26 \| 3 \| 29 \| \| - \| 78 \| 65 \| 143 \| \| Total \| 104 \| 68 \| 172 \|  \| PHB2 \| patients \| healthy \| Total \| \| --- \| --- \| --- \| --- \| \| + \| 25 \| 2 \| 27 \| \| - \| 79 \| 66 \| 145 \| \| Total \| 104 \| 68 \| 172 \|  \| RUVBL1 \| patients \| healthy \| Total \| \| --- \| --- \| --- \| --- \| \| + \| 25 \| 4 \| 29 \| \| - \| 79 \| 64 \| 143 \| \| Total \| 104 \| 68 \| 172 \|  \| RACK1 \| patients \| healthy \| Total \| \| --- \| --- \| --- \| --- \| \| + \| 32 \| 4 \| 36 \| \| - \| 72 \| 64 \| 136 \| \| Total \| 104 \| 68 \| 172 \| |
|  | Lenner et al. 1999   \| P53 \| patients \| healthy \| Total \| \| --- \| --- \| --- \| --- \| \| + \| 12 \| 3 \| 15 \| \| - \| 153 \| 227 \| 480 \| \| Total \| 165 \| 330 \| 495 \| |
|  | Liu, T et al, 2014.   \| CD25 \| patients \| healthy \| Total \| \| --- \| --- \| --- \| --- \| \| + \| 149 \| 110 \| 259 \| \| - \| 3 \| 2 \| 5 \| \| Total \| 152 \| 112 \| 264 \|  \| FOXP3 \| patients \| healthy \| Total \| \| --- \| --- \| --- \| --- \| \| + \| 151 \| 107 \| 258 \| \| - \| 1 \| 5 \| 6 \| \| Total \| 152 \| 112 \| 264 \| |
|  | Liu W et al,2015   \| IMP2/P62 \| patients \| healthy \| Total \| \| --- \| --- \| --- \| --- \| \| + \| 7 \| 1 \| 8 \| \| - \| 42 \| 43 \| 85 \| \| Total \| 49 \| 44 \| 93 \| |
|  | Liu W et al,2015   \| IMP1 \| patients \| healthy \| Total \| \| --- \| --- \| --- \| --- \| \| + \| 9 \| 0 \| 9 \| \| - \| 40 \| 38 \| 78 \| \| Total \| 49 \| 38 \| 87 \|  \| P16 \| patients \| healthy \| Total \| \| --- \| --- \| --- \| --- \| \| + \| 7 \| 0 \| 7 \| \| - \| 42 \| 38 \| 80 \| \| Total \| 49 \| 38 \| 87 \|  \| CycB1 \| patients \| healthy \| Total \| \| --- \| --- \| --- \| --- \| \| + \| 9 \| 1 \| 10 \| \| - \| 40 \| 37 \| 77 \| \| Total \| 49 \| 38 \| 87 \|  \| Survivin \| patients \| healthy \| Total \| \| --- \| --- \| --- \| --- \| \| + \| 8 \| 1 \| 9 \| \| - \| 41 \| 37 \| 78 \| \| Total \| 49 \| 38 \| 87 \|  \| Koc \| patients \| healthy \| Total \| \| --- \| --- \| --- \| --- \| \| + \| 8 \| 1 \| 9 \| \| - \| 41 \| 37 \| 78 \| \| Total \| 49 \| 38 \| 87 \|  \| c-myc \| patients \| healthy \| Total \| \| --- \| --- \| --- \| --- \| \| + \| 6 \| 0 \| 6 \| \| - \| 43 \| 38 \| 81 \| \| Total \| 49 \| 38 \| 87 \|  \| Panel \| patients \| healthy \| Total \| \| --- \| --- \| --- \| --- \| \| + \| 33 \| 3 \| 36 \| \| - \| 16 \| 35 \| 51 \| \| Total \| 49 \| 38 \| 87 \| |
|  | Liu X, 2014   \| p90/CIP2A \| patients \| healthy \| Total \| \| --- \| --- \| --- \| --- \| \| + \| 32 \| 2 \| 34 \| \| - \| 136 \| 86 \| 222 \| \| Total \| 168 \| 88 \| 256 \| |
|  | Lopez et al. 2012   \| A1AT \| patients \| healthy \| Total \| \| --- \| --- \| --- \| --- \| \| + \| 24 \| 2 \| 26 \| \| - \| 1 \| 18 \| 19 \| \| Total \| 25 \| 20 \| 45 \| |
|  | Lu H, 2012   \| Cyc B1 \| patients \| healthy \| Total \| \| --- \| --- \| --- \| --- \| \| + \| 12 \| 3 \| 15 \| \| - \| 86 \| 95 \| 181 \| \| Total \| 98 \| 98 \| 196 \|  \| HER2 \| patients \| healthy \| Total \| \| --- \| --- \| --- \| --- \| \| + \| 17 \| 6 \| 23 \| \| - \| 81 \| 92 \| 173 \| \| Total \| 98 \| 98 \| 196 \|  \| p53 \| patients \| healthy \| Total \| \| --- \| --- \| --- \| --- \| \| + \| 2 \| 0 \| 2 \| \| - \| 31 \| 45 \| 76 \| \| Total \| 33 \| 45 \| 78 \|  \| CEA \| patients \| healthy \| Total \| \| --- \| --- \| --- \| --- \| \| + \| 25 \| 13 \| 38 \| \| - \| 73 \| 85 \| 158 \| \| Total \| 98 \| 98 \| 196 \|  \| HER 2 \| patients \| healthy \| Total \| \| --- \| --- \| --- \| --- \| \| + \| 5 \| 0 \| 5 \| \| - \| 28 \| 45 \| 73 \| \| Total \| 33 \| 45 \| 78 \|  \| p53 \| patients \| healthy \| Total \| \| --- \| --- \| --- \| --- \| \| + \| 22 \| 3 \| 25 \| \| - \| 76 \| 95 \| 171 \| \| Total \| 98 \| 98 \| 196 \| |
|  | Tang et al, 2010   \| MUCI IgM \| patients \| healthy \| Total \| \| --- \| --- \| --- \| --- \| \| + \| 8 \| 12 \| 20 \| \| - \| 24 \| 35 \| 59 \| \| Total \| 32 \| 47 \| 79 \|  \| MUCI IgG \| patients \| healthy \| Total \| \| --- \| --- \| --- \| --- \| \| + \| 16 \| 13 \| 29 \| \| - \| 16 \| 43 \| 59 \| \| Total \| 32 \| 56 \| 88 \| |
|  | Tominaga et al, 2000   \| P53 \| patients \| healthy \| Total \| \| --- \| --- \| --- \| --- \| \| + \| 17 \| 0 \| 17 \| \| - \| 0 \| 50 \| 50 \| \| Total \| 17 \| 50 \| 67 \|  \| P73 \| patients \| healthy \| Total \| \| --- \| --- \| --- \| --- \| \| + \| 3 \| 2 \| 5 \| \| - \| 14 \| 48 \| 62 \| \| Total \| 17 \| 50 \| 6 \|  \| P63 \| patients \| healthy \| Total \| \| --- \| --- \| --- \| --- \| \| + \| 0 \| 0 \| 0 \| \| - \| 17 \| 50 \| 67 \| \| Total \| 17 \| 50 \| 67 \| |
|  | Tomkiel et al, 2002   \| RPA32 \| patients \| healthy \| Total \| \| --- \| --- \| --- \| --- \| \| + \| 87 \| 0 \| 87 \| \| - \| 714 \| 46 \| 760 \| \| Total \| 801 \| 46 \| 847 \| |
|  | Wang J, 2015   \| Panel \| patients \| healthy \| Total \| \| --- \| --- \| --- \| --- \| \| + \| 31 \| 2 \| 33 \| \| - \| 64 \| 93 \| 157 \| \| Total \| 95 \| 95 \| 180 \| |
|  | Wu et al, 2009   \| p53 \| patients \| healthy \| Total \| \| --- \| --- \| --- \| --- \| \| + \| 4 \| 9 \| 13 \| \| - \| 21 \| 870 \| 891 \| \| Total \| 25 \| 879 \| 904 \| |
|  | Yahalom et al, 2013   \| 13 AB panel \| patients \| healthy \| Total \| \| --- \| --- \| --- \| --- \| \| + \| 177 \| 162 \| 339 \| \| - \| 9 \| 159 \| 168 \| \| Total \| 186 \| 321 \| 507 \|   Whole population Training set Prediction set   \| 13 AB panel \| patients \| healthy \| Total \| \| --- \| --- \| --- \| --- \| \| + \| 15 \| 18 \| 33 \| \| - \| 0 \| 15 \| 15 \| \| Total \| 15 \| 33 \| 48 \|      \| 13 AB panel \| patients \| healthy \| Total \| \| --- \| --- \| --- \| --- \| \| + \| 89 \| 42 \| 131 \| \| - \| 5 \| 68 \| 73 \| \| Total \| 94 \| 110 \| 204 \| |
|  | \| p62 \| patients \| healthy \| Total \| \| --- \| --- \| --- \| --- \| \| + \| 5 \| 1 \| 6 \| \| - \| 36 \| 81 \| 117 \| \| Total \| 41 \| 82 \| 123 \|   Ye et al, 2012   \| Cyclin B1 \| patients \| healthy \| Total \| \| --- \| --- \| --- \| --- \| \| + \| 7 \| 1 \| 8 \| \| - \| 34 \| 81 \| 115 \| \| Total \| 41 \| 82 \| 123 \|  \| c-Myc \| patients \| healthy \| Total \| \| --- \| --- \| --- \| --- \| \| + \| 9 \| 0 \| 9 \| \| - \| 32 \| 82 \| 114 \| \| Total \| 41 \| 82 \| 123 \|  \| survivin \| patients \| healthy \| Total \| \| --- \| --- \| --- \| --- \| \| + \| 9 \| 1 \| 10 \| \| - \| 32 \| 81 \| 123 \| \| Total \| 41 \| 82 \| 123 \|  \| CDK2 \| patients \| healthy \| Total \| \| --- \| --- \| --- \| --- \| \| + \| 4 \| 1 \| 5 \| \| - \| 37 \| 81 \| 118 \| \| Total \| 41 \| 82 \| 123 \|  \| Cyclin D1 \| patients \| healthy \| Total \| \| --- \| --- \| --- \| --- \| \| + \| 7 \| 2 \| 9 \| \| - \| 34 \| 80 \| 114 \| \| Total \| 41 \| 82 \| 123 \|  \| Panel \| patients \| healthy \| Total \| \| --- \| --- \| --- \| --- \| \| + \| 25 \| 9 \| 34 \| \| - \| 16 \| 73 \| 89 \| \| Total \| 41 \| 82 \| 123 \| |
|  | \| Panel 3 \| patients \| healthy \| Total \| \| --- \| --- \| --- \| --- \| \| + \| 6 \| 0 \| 6 \| \| - \| 125 \| 135 \| 260 \| \| Total \| 131 \| 135 \| 266 \|   Zhu et al, 2015   \| BRCA2 \| patients \| healthy \| Total \| \| --- \| --- \| --- \| --- \| \| + \| 48 \| 1 \| 49 \| \| - \| 83 \| 134 \| 217 \| \| Total \| 131 \| 135 \| 266 \|  \| PARP1 \| patients \| healthy \| Total \| \| --- \| --- \| --- \| --- \| \| + \| 20 \| 1 \| 21 \| \| - \| 111 \| 134 \| 245 \| \| Total \| 131 \| 135 \| 266 \|  \| BRCA1 \| patients \| healthy \| Total \| \| --- \| --- \| --- \| --- \| \| + \| 25 \| 1 \| 26 \| \| - \| 106 \| 134 \| 240 \| \| Total \| 131 \| 135 \| 266 \|  \| Panel 4 \| patients \| healthy \| Total \| \| --- \| --- \| --- \| --- \| \| + \| 6 \| 0 \| 6 \| \| - \| 125 \| 135 \| 260 \| \| Total \| 131 \| 135 \| 266 \|  \| Panel 1 \| patients \| healthy \| Total \| \| --- \| --- \| --- \| --- \| \| + \| 7 \| 0 \| 7 \| \| - \| 124 \| 135 \| 259 \| \| Total \| 131 \| 135 \| 266 \|  \| Panel 2 \| patients \| healthy \| Total \| \| --- \| --- \| --- \| --- \| \| + \| 10 \| 0 \| 10 \| \| - \| 121 \| 135 \| 256 \| \| Total \| 131 \| 135 \| 266 \| |
|  | Zuo et al, 2014   \| Panel \| patients \| healthy \| Total \| \| --- \| --- \| --- \| --- \| \| + \| 96 \| 13 \| 109 \| \| - \| 14 \| 42 \| 56 \| \| Total \| 110 \| 55 \| 165 \| |
