# Supplementary figures and images for: Can serum autoantibodies be a potential early detection biomarker for breast cancer in women? A diagnostic test accuracy review and meta-analysis
Source: Syst Rev. 2022 Oct 9;11:215. doi: 10.1186/s13643-022-02088-y (PMC9549667; doi:10.1186/s13643-022-02088-y)

**Summary ROC (SROC) curves of autoantibodies**

1. P53

**
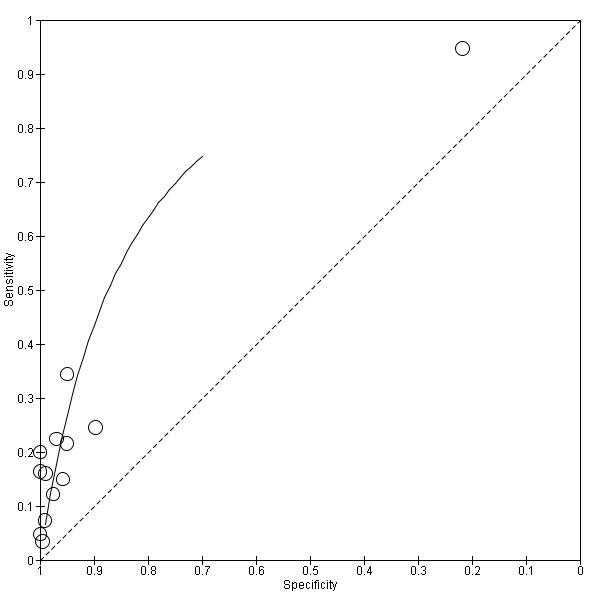
**

1. MUC1

**
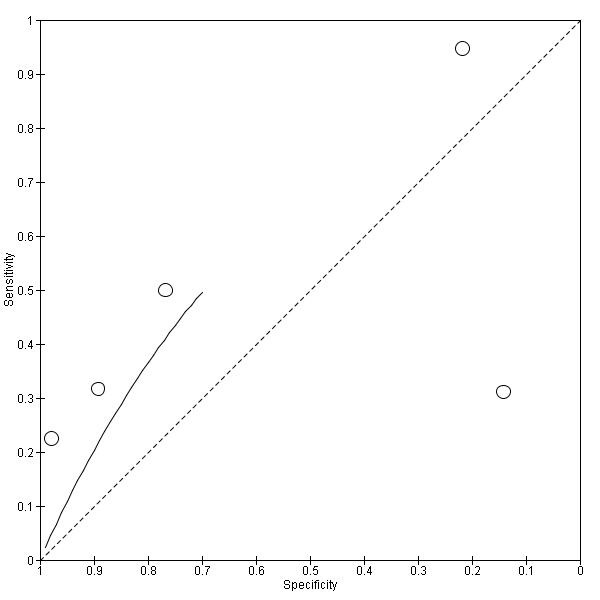
**

1. HER2


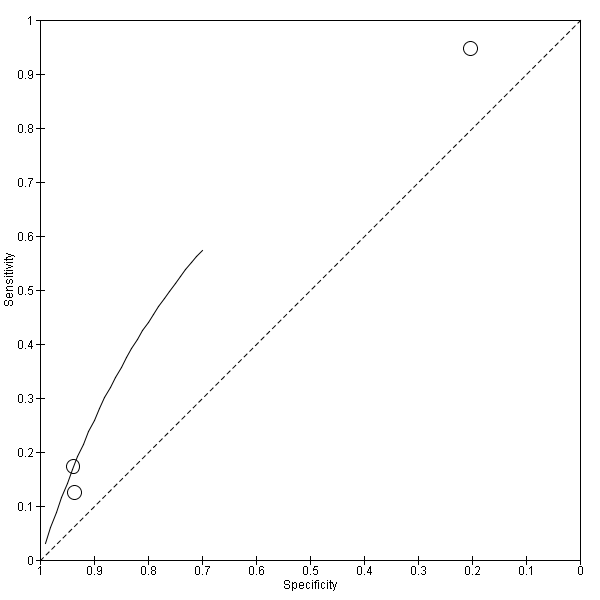


1. HSP60


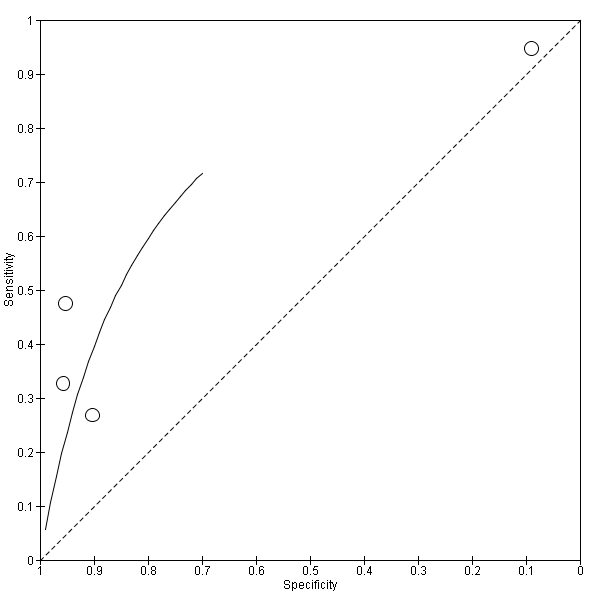


1. P16


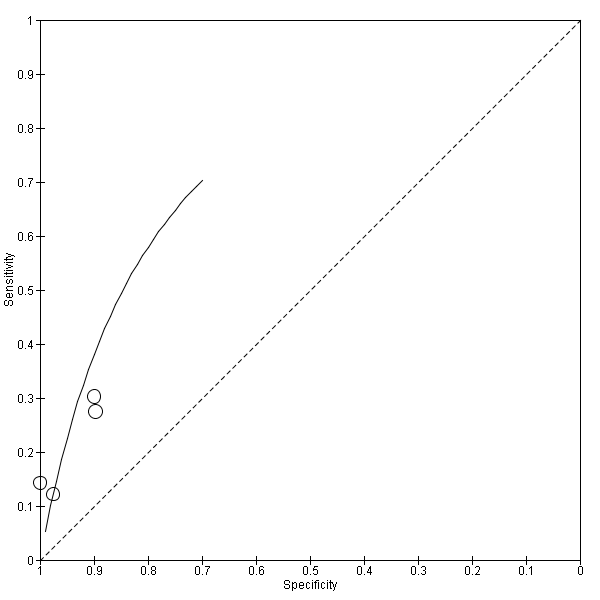


1. Cyclin B1


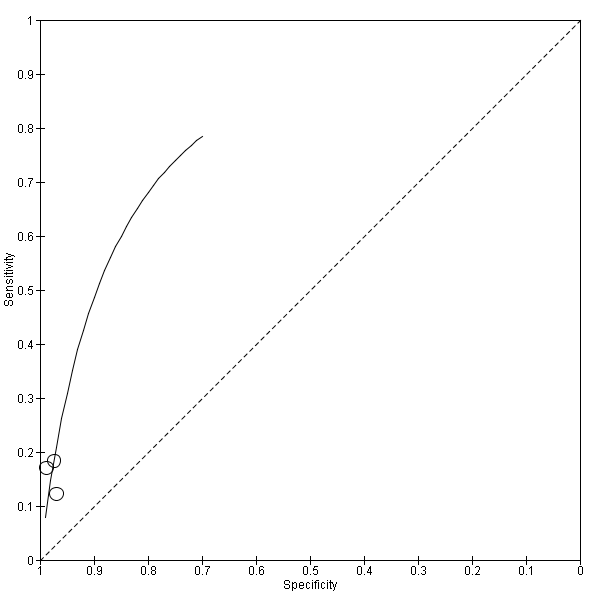


1. c-Myc

**
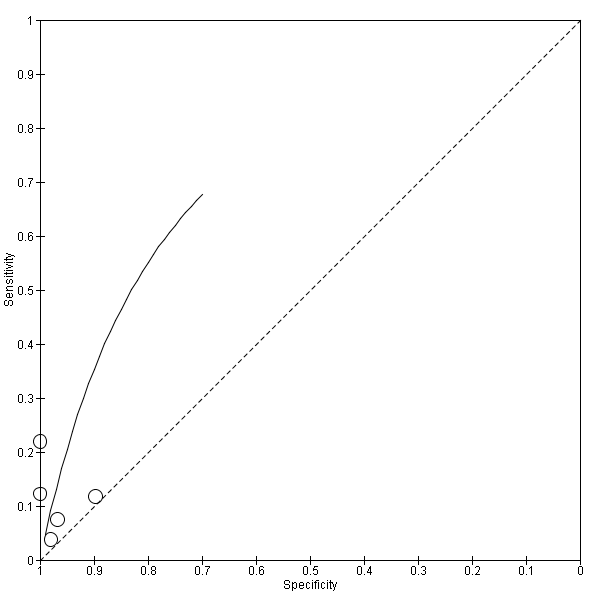
**

Supplement: Supplementary file 9 — Additional file 9. Summary ROC (SROC) curves of autoantibodies. [file 13643_2022_2088_MOESM9_ESM.docx]
